# Investigating identification disparities in forensic anthropology casework

**DOI:** 10.1371/journal.pone.0290302

**Published:** 2023-11-01

**Authors:** Cris Hughes, An-Di Yim, Chelsey Juarez, John Servello, Richard Thomas, Nicholas Passalacqua, Angela Soler

**Affiliations:** 1 Department of Anthropology, University of Illinois at Champaign-Urbana, Urbana, Illinois, United States of America; 2 Carl R. Woese Institute for Genomic Biology, University of Illinois at Champaign-Urbana, Urbana, Illinois, United States of America; 3 Department of Health and Exercise Sciences, Truman State University, Kirksville, Missouri, United States of America; 4 Forensic Science Program, George Mason University, Fairfax, Virginiai, United States of America; 5 Department of Anthropology, California State University Fresno, Fresno, California, United States of America; 6 Forensic Anthropology Unit, University of North Texas Center for Human Identification, Fort Worth, Texas, United States of America; 7 Trace Evidence Unit, Laboratory Division, Federal Bureau of Investigation, Quantico, Virginia, United States of America; 8 Anthropology and Sociology Department, Western Carolina University, Cullowhee, North Carolina, United States of America; 9 New York City Office of Chief Medical Examiner, New York City, New York, United States of America; University of Macerata: Universita degli Studi di Macerata, ITALY

## Abstract

Forensic anthropology is shifting to reflect on the impact of its practices within the criminal justice context in important ways. Here, we contribute to this essential work by examining how decedent demographics as well as estimations of biological profile components are related to identification trends in forensic anthropology cases. The study uses data from more than 1,200 identified and unidentified forensic anthropology cases from three agencies (together representing a nation-wide sample). We found the following: i) multivariate analyses indicated that decedent sex, age, and race and/or ethnicity are not related to case identification rates in the pooled United States sample, ii) when identification rate differences do occur, they appear to be smaller effects, more agency-specific, and/or related to the context of a particular agency, iii) for the agency-specific sample with available data, there was no consistent evidence for a discrepancy in the duration of an identification investigation based on a decedent’s sex, age, or race and/or ethnicity, iv) forensic anthropological estimations of sex, age, and ancestry can improve the odds of identification for decedents, although these are small effects, and v) reporting an ancestry estimation does not appear to impact decedent race representation among resolved unidentified person cases. Although previous studies have identified demographic discrepancies in other areas of the criminal justice system, the results presented here suggest that decedent demographic estimation practices by forensic anthropologists in general do not appear to be related to discrepancies in identification trends, but more research is needed to examine whether these findings hold. Contextual factors and practices specific to each investigative agency likely contribute to identification trends.

## Introduction

With the National Academy of Science’s report, *Strengthening Forensic Science in the United States*: *A Path Forward* over a decade in the rearview mirror, research in forensic anthropology has consistently and robustly addressed measures for assessing the accuracy and reliability of approaches to forensic casework. While the standards laid out in that report will continue to inform methodological approaches and research in forensic anthropology for years to come, recent discussions and work in the discipline have highlighted the need to give equal consideration to researching the *contextual* impacts of the practice of forensic anthropology—in other words, discovering and coming to terms with both positive and deleterious downstream effects of forensic anthropology practice in the investigation context, as well as the broader social context.

In the summer of 2020, nationwide demands for radical change to the criminal justice system were ignited as a result of the high profile (but not isolated) incidents of a series of police killings of Black individuals in the United States. Within the discipline of forensic anthropology, researchers simultaneously called for the critical examination of ancestry estimation as it relates to concepts and applications of race in research and unidentified persons casework. Specifically, Bethard and DiGangi [1] asserted that the practice of ancestry estimation of unidentified human skeletal remains by forensic anthropologists is doing harm to communities of color by reifying racialized science, hindering identifications in casework, and applying methods with skeletal data for which we lack evolutionary understanding. In response, Stull and colleagues [2] countered that ancestry estimation plays a role in repatriation and determination of medicolegal significance and cautioned against abandoning ancestry estimation without consultation with both stakeholders and anthropologists in the discipline. While providing different viewpoints, both Bethard and DiGangi [1] and Stull et al. [2] did align regarding the need for data-driven research to investigate each others’ proposed perspectives. This conversation garnered national attention and laid the groundwork for recent critical discussions and research on issues of diversity and inclusion [3–6], the perception and framing of race and ancestry [7–12], as well as gender and biological sex [13–15], and reexamination and improvement of the language used in our research [16, 17]. As forensic anthropology works to critically engage with concepts beyond the science itself and associated methodological approaches, it is essential to recognize that this discipline is part of the larger community of death investigation, for which similar conversations are rarely happening (with a few exceptions, see [18, 19]), yet are equally necessary. Forensic anthropologists’ work at self-criticality has implications not only for our own practice, but also has the potential to impact and improve the practice of the greater medicolegal death investigation community [20].

One goal of forensic anthropological analyses in medicolegal death investigations is to produce information from an unidentified person’s skeletal remains that narrows down the pool of potential missing persons from which this person may have originated, thus producing an investigative lead that may assist in the successful identification of the person. Forensic anthropologists engage with socially-constructed concepts of race and ethnicity because these are employed as criteria used to narrow the pool of potential matches within the missing and unidentified person investigation context in the U.S. [2, 21]. Furthermore, tracking decedent race and ethnicity allows researchers to provide insight into decedent trends. Recent studies have provided forensic anthropology with a clearer sense of the demographics of decedents common in forensic anthropology cases where the decedents were successfully identified [22, 23]. Notably, research indicated that Black and Hispanic persons were disproportionately overrepresented in forensic anthropology casework when compared to U.S. census data population proportions. While insight into demographic representation is an important first step, it has been difficult to comprehensively track investigative trends that may produce and/or result in investigative outcome disparities (e.g. successful identification rates) across demographics for forensic anthropology casework. Researching investigative trends, strategies, hurdles, and biases—all of which may differ at an agency level and can influence the investigative success of an unidentified persons case—include myriad factors for which readily available, standardized data do not exist. However, given that these factors influence investigative outcomes (e.g. duration of an investigation to completion; successfully making an identification), exploring whether there are trends and/or disparities in these outcomes is a logical first step. Here, we explore such investigative outcomes within the context of decedent demographic factors of sex, age, and race/ethnicity, and the information provided by the forensic anthropologist, using available data on casework success and investigation duration. Specifically, this study addresses the following:

Aim 1. *For forensic anthropology cases*, *do investigation trends (e*.*g*. *identification success rate; duration of an investigation) differ across demographic backgrounds (e*.*g*. *sex*, *age*, *race/ethnicity)*? Based on the extensive bias and structural violence and vulnerabilities documented in the criminal justice system and forensic investigations (discussed in depth below), we hypothesize that Black, Indigenous, and People of Color (BIPOC) cases may have lower success rates or longer investigative durations for identifying the skeletal remains as a missing person. In the present study, our first aim is to test this hypothesis using decedent demographic data from over 1,200 U.S. forensic anthropology cases of unidentified persons (both resolved and open).
Aim 2. *Is the decedent information included in forensic anthropology reports (e*.*g*., *estimations of decedent ancestry*, *sex*, *and age) related to the (de)prioritization of particular individuals over others in regard to identification efforts*. This aim corresponds to the hypotheses proposed by Bethard and DiGangi [1] that information related to decedent race/ethnicity (e.g. forensic anthropologists’ ancestry estimations) of an unidentified person may provide grounds for “racial bias on the part of investigators” which “may hinder identification efforts.” Indeed, they propose that the very information provided by forensic anthropologists may be a driving factor for inadequate efforts by investigators to resolve cases of non-White decedents [1]. While data that directly correspond to prioritization by investigators are unavailable in the present study, proxy data are employed, including the duration of an investigation as well as success rates of identifications. Given that Bethard and DiGangi [1] hypothesize that investigations may be hindered when relevant demographic information is provided to investigators, investigative success and duration are proxies that represent the comprehensive investigation. Importantly, investigation hinderances and/or deprioritizations would be included in the factors influencing investigative success and duration, and if sufficient to influence the investigation would be observable in the present analysis. Bethard and DiGangi’s hypothesis is explored in several ways, using data on ancestry estimation availability, and when estimated, what those corresponded to in terms of race/ethnicity. The analysis was further expanded to include both age and sex estimations, to comprehensively explore how forensic anthropologists’ estimations of demographic data influence an investigation.
*Aim 3*. *Does providing information related to the decedent’s age*, *sex*, *and/or race/ethnicity influence the odds of an identification*? For this research aim, the hypothesis is based on Bethard and DiGangi [1], who challenge the assumption that ancestry estimations are critical for contributing to successful investigations of unidentified persons. We extend the testing of this assumption beyond ancestry estimations to include estimations of age and sex. We compare the identification rate for cases with and without these estimations.

Documenting decedent demographic and investigative trends in United States forensic casework allows for a better understanding of how institutional (i.e., United States Criminal Justice System) and societal factors may be related to, or directly affecting the time to identification and the overall success of identification for various populations within the United States. Thus, documenting demographic information and analyzing these data for trends can in turn have important implications for improving approaches to identification investigations [24–26].

It is worth noting that forensic anthropological analyses typically represent a small component of identification investigations, and demographic and investigative trends potentially vary depending on which aspect of the forensic setting one examines (e.g., all medicolegal death investigations *versus* forensic anthropology cases). However, the goal of this study is to explore whether there is a relationship between identification status of decedents who receive an anthropological analysis and demographic factors (including sex, age, and race and/or ethnicity) to infer whether any differences in case identifications exist at a regional and/or national scale.

### Forensic anthropology’s discourse on ancestry estimation and race

The present focus on examining whether investigation and identification trends differ among decedent demographic groups stems from the much broader discourse within the discipline of forensic anthropology. The broader discourse was borne out of the initial work of Bethard and DiGangi [1], in which the authors called on the U.S. forensic anthropology community to stop conducting ancestry estimations in forensic anthropology casework for three main reasons. First, they insisted forensic anthropologists have not considered whether ancestry estimations might hinder identification efforts, related to the entrenched (conscious and unconscious) racial biases within the criminal justice system at large. That is, for example, unknown human remains whose ancestry estimation is consistent with the decedent’s race being Black or African American (but potentially extends to BIPOC individuals as a whole) may receive less investigative attention/prioritization than those decedents considered White. Secondly, the authors proposed a framework in which the practice of ancestry estimation reifies race as biology and perpetuates typological notions of race. Essentially, they argued that the act of ancestry estimation itself, the categorizing of humans based on phenotypes is in and of itself the “reliving” of a practice deeply imbedded in a racist history which supported not only slavery, the divestment of Native Peoples from their land, and Jim Crow, but is allowed to continue because of the lack of a reckoning with biological anthropology’s racialized past [27, 28]. Thirdly, Bethard and DiGangi [1] demand a cessation to the use of macromorphoscopic traits in ancestry estimation without a full grasp of the heritability of these traits.

In response to the initial publication by Bethard and DiGangi (1), scholars and practitioners have begun to open up the conversations to an array of relevant findings. Adams and Pilloud (7) explored instances of misappropriation of psychology and biological anthropology research by white nationalists to justify their racist beliefs, and documented ways in which biological anthropology research was used to argue for the existence of racial typology and urged researchers to consider the broader implications of their research beyond their discipline and intended application. Other recent works [10, 11, 29–32] recommend moving towards a practice of estimating population affinity, which advances beyond continental allocations often associated with ancestry estimations, and incorporates a comprehensive understanding of population histories as well as ongoing dynamics (sociopolitical, economic) that can influence local patterns of biological variation. In turn, this approach considers how categories and terms used to describe the reference samples represent social meaning relevant to local communities [17, 30, 31]. Several studies propose that current methodological approaches and reference sample representation may be impacting the accuracy rates of ancestry estimation, and suggest that increasing diversity of reference samples would better serve the forensic anthropological casework demographics [22, 23]. Furthermore, Go and colleagues [33] documented how those conducting ancestry-related research, as well as the skeletal samples used, are largely WEIRD (Western, educated, industrialized, rich, and democratic). The authors rightly pointed out that this lack of diversity among practitioners and samples reifies harm and “informs the history, values, and practices of forensic anthropology on a global scale” (Go et al., [33], p. 156).

### Established demographic trends in U.S. investigations

The previously-reviewed body of work provides data points forensic anthropologists can use to inform their decision (both collectively and individually) of whether and how they will practice ancestry or population affinity estimation moving forward. The current study provides another “data point” for consideration in the larger ongoing conversation regarding the contextual impacts of our practice. Furthermore, this study intentionally expands the inquiries beyond ancestry estimation, to incorporate estimations of sex and age, as research has established that other demographic factors in addition to or in tandem with race/ethnicity are related to investigation and criminal justice disparities. Bethard and DiGangi [1] and DiGangi and Bethard [34] referenced sociological work on “missing White woman syndrome” as an example, which highlights how certain cases (those involving White women) are prioritized in social media and news outlets over others [35] and therefore have greater odds of resolution. Other studies have explored the extent to which BIPOC individuals and/or members of the LGBTQ+ community are negatively impacted by the criminal justice system in a variety of ways, from police engagements, to investigative outcomes, to sentencing and parole results [36–44]. Tallman et al. [15] surveyed 128 forensic anthropologists and found 28.9% had encountered transgender individuals as part of their casework and called for greater consideration of queer theory in forensic anthropology research and practice.

Within the medicolegal death investigation system, Goliath and Cosgriff-Hernandez [39] comprehensively reviewed racial disparities and biases, including the criminal justice system policies that have led to a disproportionate number of BIPOC individuals and adults coming from vulnerable circumstances represented in the system. Studies have suggested that investigations of missing Black individuals remain unresolved more often, and when resolutions do occur, take longer than cases involving White individuals [45, 46]. This statistic is particularly concerning as we know that Black individuals make up a disproportionate number of missing and unidentified cases in the United States at 33%, yet only comprise approximately 13% of the United States’ population [47]. Related to this discrepancy, it has been argued that differential treatment of various demographic groups has ultimately influenced who is comfortable and/or willing to engage with law enforcement regarding investigations, including investigations of unidentified remains and missing persons [48–53].

Based on the works reviewed here, scientific investigation is warranted regarding whether demographic factors are related to identification status (identified or not) and the rate (i.e., death investigation duration) at which a case is resolved. If such disparities exist, the reasons can be multifactorial, such as investigative practices, practitioner choices, public participation and/or trust in investigations, and structural vulnerabilities. Collectively, the works cited above suggest that there is much more to understand regarding decedent demographic trends related to forensic anthropology practice. By studying the relationship of decedent demographics and identification trends for forensic anthropology casework, we can begin to understand the complex roles that forensic anthropologists’ estimation of a decedent’s biological profile (including sex, age, and ancestry) may have on the identification process. The results of this study can help inform practitioner strategies and policies for mitigating investigation biases.

#### Addressing analytical limitations: Data and interpretations

It is important to note that if demographic differences in case identification trends are found in this study, it does not provide evidence for a direct correlation to overt prioritization of one case demographic over another; however, it would provide a foundation and direction for further study into what may be driving such differences. Concomitantly, if demographic differences in case identification trends are *not* found, it does not provide evidence that medicolegal death investigations are wholly unbiased. Because case investigations (and their success or failure) depend on myriad factors, this study is only a first step in exploring identification trends related to decedent demographics in United States forensic anthropology casework.

It is important to set expectations of studies with case datasets like this up front, and it is helpful to consider comparable roadblocks highlighted in criminal justice research. Knox and Mummolo [54] examined why research on whether and to what degree police behavior is racially biased was not as forthcoming and conclusive as one might hope, given the heightened attention by scholars in recent years. They emphasize that data constraints/limitations only allow the study of isolated aspects of police–civilian encounters, and thus do not fully represent the myriad of choices, interactions, standard operating procedures, and other factors related to case contexts. Indeed, the data being relied upon by researchers are largely generated by police for police administrative purposes, ultimately producing a fragmentary data source that can lead to incomplete and disparate results when analyzed, given that the original data-generation was not geared towards answering the kind of questions that are now in the forefront of many social scientists’ minds. Relating to the present study, we acknowledge that medicolegal death investigation data of forensic anthropology cases largely originates with the same purposes, for case management and tracking, and is bound by all of the nuanced choices of what to track (and what not to track) and how to track it. If disparities in identification trends exist, then it would be ideal to explore elements of an investigative context that could produce such disparities, such as identification modality, availability of missing person reports/antemortem records/family reference DNA samples, and the quality of evidentiary data, and more. Here, we initiate the work for establishing whether investigative disparities for forensic anthropology cases exist, but the exhaustive factors that may be producing disparities are not readily available in this study. For this initial study, we focus on decedent sex, age, race, as well as agency, case year, and geographic location.

## Materials and methods

### Materials

The forensic anthropology unidentified persons cases used in this study were drawn from three agencies’ databases: the Federal Bureau of Investigation Laboratory, the New York City Office of Chief Medical Examiner (NYC OCME), and the University of North Texas Center for Human Identification (UNTCHI) Forensic Anthropology Unit (FAU). Table 1 provides data for the three agencies regarding sample sizes, the case year ranges, and the percent of individuals identified in each agency sample. Both resolved (i.e. person is identified) and unresolved (i.e. persons’ remains unidentified to date) cases were included. Below is a brief description of any additional considerations for each agency dataset. To provide consistency in casework across the three agencies, cases were included in the sample if they met all of the following criteria: the remains were decomposed beyond the fresh stages, estimation of the biological profile was completed, no tentative, presumed, or positive identification was known upon case intake, and cases were determined to be of medicolegal significance. Tentative identification data were inferred from the case investigation notes or directly reported from the investigating agency. For resolved cases, many had comprehensive information on the individuals’ demographic data for sex, age, and race and/or ethnicity, however some were missing information which is why the sample sizes vary per analysis performed.

**Table 1:** Agency sample details.

	Sample size (total)	Case Year Range	% Cases Identified
**FBI**	524	1964–2021	41%
**NYC**	368	1989–2021	57%
**UNT**	361	2008–2020	44%
**Total**	1253	1964–2021	47%

#### FBI case dataset

The Federal Bureau of Investigation (FBI) Laboratory performs a broad range of forensic examinations on skeletal remains and maintains detailed files for a large number of resolved and unresolved unidentified decedent cases submitted by jurisdictions nationwide. These case files often contain an anthropological biological profile assessment, including estimates of age, sex, ancestry and stature. For those cases with successful positive identification of the decedents via DNA analysis or other investigative means, the biological profile characteristics of the decedents are known and documented. These cases contain anthropological examinations conducted both by anthropologists at the FBI Laboratory and outside of the FBI Laboratory, providing a variety of analyses and data sources. Overall, 525 cases (with most anthropological examinations dating from 2000 or later) were selected. In general, cases with older anthropological examinations were submitted to the Laboratory for DNA analysis, which was not available at the time of recovery, or for facial approximation as part of cold case initiatives carried out by local medicolegal authorities. Reported sample variables used in the present study include: case recovery and analysis years; case identification status (identified or not identified); forensic anthropological estimates of sex, age, and ancestry for the decedents; known decedent demographic data (sex, age, race); and the National Missing and Unidentified System’s (NamUs) region code for case origin. Of note for this study, a key feature of the FBI dataset is that the vast majority of the forensic anthropology cases were investigated by an outside agency with jurisdiction over the case, not the FBI. Therefore, the FBI’s involvement in these cases is limited to the services requested from the FBI Laboratory by the outside agency and does not include ongoing investigation of these cases. In this study we will refer to the FBI agency dataset, but it is necessary to recognize that the FBI’s dataset represents cases worked by forensic anthropologists from an array of agencies and that the FBI does not have jurisdiction over these cases.

#### NYC case dataset

This portion of the study dataset is comprised of resolved and unresolved cases (n = 368) from the New York City Office of Chief Medical Examiner (NYC OCME). For those cases with positive identification of the remains, the biological profile characteristics of the decedents are known and documented. These cases span several decades, with the majority of cases with forensic anthropological analyses carried out from 2004 onward, but some of the cases prior to this have been retroactively examined by forensic anthropologists at the agency. The majority of cases in this sample are from the New York City jurisdiction, but some are from other agencies in the surrounding areas within New York State. Thus, the originating agency for the majority of the NYC dataset is the same as the agency performing the forensic analysis and investigation of the cases, which is very different from the situations described for UNT and FBI. Reported sample variables used in the present study included: case recovery and analysis years; case identification status (identified or not identified); forensic anthropological estimates of sex, age, and ancestry for the decedents; known decedent demographic data (sex, age, race); and the NamUs region code for case origin.

#### UNT case dataset

This portion of the study dataset consists of resolved and unresolved cases (n = 361) examined at the University of North Texas Center for Human Identification (UNTCHI) Forensic Anthropology Unit (FAU) from 2008 to 2020. Remains examined at the FAU were submitted by investigative agencies nationwide. Cases were recovered between 1960 and 2020, the majority of which were found between 2012–2018. For those cases with positive identification of the remains via DNA analysis or other investigative means, the biological profile characteristics of the decedents are known and documented. Additionally, cases with positive molecular associations (i.e., “DNA identifications”) were coded for presence/absence of available reference profiles (Family Reference Samples or CODIS-Convicted Offender Index). Reported sample variables used in the present study included: case recovery and analysis years; case identification status (identified or not identified); identification year; forensic anthropological estimates of sex, age, and ancestry for the decedents; known decedent demographic data (sex, age, race); and the NamUs region code for case origin. A key feature of the UNT dataset is that it does not have jurisdiction over the cases they examine because they are from other investigative agencies that are outsourcing the forensic anthropology analysis to UNT. Thus, UNT’s involvement in these cases is limited to the services requested by the originating entity and is not directly involved in the ongoing investigation of these cases. In this study we will refer to the UNT agency dataset, but it is necessary to recognize that UNT’s dataset represents cases worked by forensic anthropologists at UNT, however UNT is not the originating agency for any of these cases.

#### Case data standardization

In the dataset, there is both known and estimated demographic information about the decedents. *Known* demographic information is derived from resolved cases when the decedent is positively identified, and includes sex, age and race/ethnicity often derived from legal or medical documentation/records. Not all resolved cases (i.e. identified cases) contain sex, age and race/ethnicity information, as these may not be known or available to the agency at the time of case resolution. *Estimated* demographic information is derived from the forensic anthropological analysis of the skeletal remains, and includes the estimation of biological sex, age, and ancestry. Not all resolved or unresolved cases have estimated demographic data, due to limitations with the analyses such as damaged or missing skeletal elements used by forensic anthropologists for these estimations. Both known and estimated demographic data are used separately or in conjunction as appropriate per study analysis. These are described in detail in the Supporting Information.

### Methods

All but multivariate analyses were performed using JMP^®^ Pro 16.1.0. Multivariate modeling fitting was performed using the ‘lme4’ package [55] in the R Programming Language 4.1.0 [56]. Initial univariate comparisons of the forensic anthropology caseloads from the three agencies (UNT, FBI, NYC) were completed, including a comparison of the NamUs region of origin and the decedent demographic data distributions for each agency (sex, age, and race and/or ethnicity). Chi-square tests for agency distribution differences were carried out to compare the three samples. Identification status (identified or not identified) trends were then generated and compared across decedent demographic variables, and Chi-square tests (or Fisher’s Exact test when appropriate) and correspondence analyses were completed to compare the demographic distributions of identified versus unidentified individuals. We also examined the availability of biological profile information as it relates to identification rates.

Wilcoxon Rank Sum/Kruskal Wallis tests were used to infer whether there were significant differences in investigation duration (length of time to identification in years) across decedent demographic groups. This analysis requires an identified case sample and was completed using the UNT sample because both recovery date (by year) and identification date (by year) were only available from this agency. For the UNT identified case sample, the “investigation duration” was determined to be the total number of years from case recovery to identification.

Importantly, the analyses of investigation duration would inevitably include cold cases from many decades ago, and the prevalence of such cases will be dependent on the trends in U.S. demographics over the years, which could bias the sample if left unchecked and produce substantially different mean investigation durations. For example, while there are substantial cases in our dataset representing White decedents from 20 to 50 years ago, the majority of cases representing Hispanic decedents occur in more recent decades. As another example, improvements to investigations in more recent decades could produce shorter investigative durations. In order to mitigate the effects of these factors on identification duration, we took the following steps. For the identified case analysis of the relationship between investigation duration and decedent race, only Hispanic and White had sufficient sample sizes for comparison. Based on the case recovery year distributions, we found that Hispanic decedent cases became more prevalent in the UNT sample more recently, especially over the past decades, with the 90% (10% quantile) of all UNT Hispanic cases occurring in or after 2012, and the 75% (25% quantile) occurring in or after 2014. These were therefore used as the case year cutoffs in order to ensure comparable year distributions and prevalence for the White and Hispanic decedents. Furthermore, this time period allows for comparable identification modalities, as all cases are included in the period when UNT had DNA analyses regularly integrated into casework.

Mixed effects logistic regressions were employed to examine i) combinations of sex, age, and race and/or ethnicity as they relate to identification status, and ii) whether having estimates of biological profile components influences identification status. A mixed effects model taking into account the non-independence in the dataset was employed. In this case, we assume there are agency-specific identification trends (with regards to demographic factors) in addition to general trends in identification. That is, cases from one agency were handled by a particular group of practitioners with agency-specific training and experience and may be subject to the same influences. It is therefore necessary to explore agency-specific trends using a multivariate approach. Identification status was modeled as a binary outcome variable (identified vs. unidentified) while sex, age, race and/or ethnicity, and whether these components were estimated were modeled as categorical predictor variables. To ensure a fully represented data matrix, the combined demographic categories with zero cases were removed prior to model fitting. Since doing so removed all Juvenile individuals and all Native American individuals, we report both univariate and multivariate analyses results as they complement one another and provide a more comprehensive picture of the identification trends. The resulting model was then checked for collinearity between predictors as well as influential cases to ensure model assumptions are met [57]. To minimize abstraction, we describe our models in terms of comparison with the reference categories for the predictors. The reference categories are females, adolescents, and White.

Because multiple tests were performed on the same subsamples of the dataset, we used the Benjamini-Hochberg false discovery rate *p-*value adjustment for multiple tests [58]. To measure the effect size of the Chi-square tests, Cramer’s *V* was employed, using the 95% confidence interval for its estimated value. Cramer’s *V* is an extension of the correlation coefficient used for 2 x 2 contingency tables and ranges from zero to one, with zero implying no correlation (i.e., negligible effects) and values closer to one implying a strong correlation (i.e., large effects). Cramer’s *V* values can be interpreted using Cohen’s *d* degrees of freedom-informed approach [59]. While effect size is not traditionally used in forensic anthropology research, it has been considered a statistical best practice in many fields, including psychology, medicine, ecology and evolutionary biology [60]. However, the thresholds for interpreting what size effect is considered important is known to be relatively arbitrary unless there are other studies to compare it to. When comparative effect sizes are unavailable in the discipline, Cohen [59] proposed general thresholds for use: negligible effects (*V* ≤ 0.10), small effects (0.10 < *V* < 0.30), medium effects (0.30 < *V* < 0.50), and large effects (*V ≥* 0.50). For this study, the thresholds are defined using the ranges provided above. We report effect size for three reasons. First, given the large sample sizes used in this study, the odds of statistically significant tests at *p* = 0.05 are extremely high, but not meaningful beyond that a relationship exists (slim chance of a Type I error), but the *magnitude* of the relationship between the variables of interest cannot be directly inferred from the *p-*value [61, 62]. Thus secondly, reporting effect sizes for each of our analyses in the present study allows us to compare the strength of the relationships for each test and across agencies. Third, the effect sizes reported here can be used as the first comparison for future studies using effect sizes in our discipline when studying similar topics.

## Results

### Comparing agency datasets: Demographics, geographic distributions, and identification rates

The three agencies have substantial differences in their regional representation (Table 2), with the FBI sample being the most representative of the nine NamUs regions. The UNT sample is primarily represented by Region 4 (73% of its forensic anthropology caseload), but does have cases from all but Regions 8 and 9. In contrast, 100% of the NYC sample is from Region 6, the majority from New York City. When all three agency samples are combined, all NamUs regions are represented, although not evenly (e.g., Regions 4 and 6 comprise 54% of the total sample).

**Table 2:** Regional distribution of casework for the three agencies. Region 1 (AZ, CO, HI, NM, NV, UT, Guam, Saipan), Region 2 (AK, ID, MN, MT, ND, OR, SD, WA, WY), Region 3 (IA, IL, IN, KS, MO, NE, OH, WI), Region 4 (AR, LA, OK, TX), Region 5 (AL, KY, MS, PA, TN, WV), Region 6 (CT, MA, ME, MI, NH, NY, RI, VT), Region 7 (FL, GA, NC, SC, Puerto Rico, US Virgin Islands), Region 8 (DE, MD, NJ, VA, District of Columbia), Region 9 (CA).

Case Origin: Region	FBI Sample		NYC Sample		UNT Sample	
	%	n	%	n	%	n
Region 1	27%	140	0%	0	13%	48
Region 2	8%	42	0%	0	3%	10
Region 3	7%	35	0%	0	5%	18
Region 4	3%	14	0%	0	73%	262
Region 5	12%	63	0%	0	1%	5
Region 6	5%	24	100%	368	2%	8
Region 7	26%	135	0%	0	3%	10
Region 8	12%	65	0%	0	0%	0
Region 9	1%	6	0%	0	0%	0
All	100%	524	100%	368	100%	361

Comparisons of the distributions for decedent sex, age, and race and/or ethnicity are presented in Table 3. Regarding decedent sex, all agencies had at least twice the frequency of male decedents compared to female decedents. The FBI and NYC samples had comparable proportions, while UNT presented a slightly greater proportion of male decedents compared with the other two agencies. Age trends indicated that the UNT sample more frequently included young adults and less frequently included older adults compared to the NYC and FBI samples. When considering the combined frequencies of the three agency samples, juvenile and adolescent cases were the least common, while young adults and middle-aged adults were comparable in frequency. The greatest frequency differences in decedent demographic trends among the three agencies were for decedent race and/or ethnicity. The majority of UNT’s sample was comprised of Hispanic (55%) decedents, followed by White (17%) decedents. In contrast, the majority of NYC’s forensic anthropology sample was comprised of White (35%) and Black (25%) decedents. The majority of FBI’s sample was comprised of White individuals (43%), which were about two to three times as common as any other decedent race and/or ethnicity. For all agencies, Native American decedents and Asian decedents were the least frequent (0 to 5%).

**Table 3:** A comparison of the decedent demographic and case identification details of the three agencies.

	FBI Sample		NYC Sample		UNT Sample		Pooled Sample
	%	n	%	n	%	n	%
**Decedent Sex**							
Female	33%	171	29%	108	19%	67	28%
Male	65%	339	68%	250	72%	261	68%
Inconclusive	3%	14	3%	10	9%	33	5%
All	100%	524	100%	368	100%	361	100%
**Decedent Age**							
Adolescent	10%	52	8%	29	7%	25	8%
Juvenile	0%	2	4%	13	1%	5	2%
Young Adult	28%	147	27%	98	41%	149	31%
Middle Adult	33%	175	23%	86	32%	115	30%
Older Adult	19%	102	29%	107	7%	27	19%
Inconclusive	9%	46	10%	35	11%	40	10%
All	100%	524	100%	368	100%	361	100%
**Decedent Race and/or Ethnicity**							
Asian	3%	14	5%	18	1%	3	3%
Black	18%	96	24%	90	4%	15	16%
Hispanic	12%	61	14%	52	55%	199	25%
Native American	3%	16	0%	0	1%	2	1%
White	43%	227	35%	128	17%	62	33%
Additional Races and/or Ethnicities	0%	2	3%	11	0%	0	1%
Inconclusive	21%	108	19%	69	22%	80	21%
All	100%	524	100%	368	100%	361	100%
**Identification Status**							
Identified	41%	215	57%	211	44%	202	47%
Not Identified	59%	309	43%	157	56%	159	53%
All	100%	524	100%	368	100%	361	100%

Chi-square tests of agency-specific differences for the frequency distributions of decedent sex, age, and race and/or ethnicity were performed, excluding those cases with an inconclusive decedent sex, age, or race and/or ethnicity. Furthermore, the Native American subsample was excluded from the Chi-square test for race and/or ethnicity because the sample sizes were not substantial enough and left some cells with zero cases. All tests for agency differences in decedent sex, age, and race and/or ethnicity frequencies yielded statistically significantly results (Table 4). The accompanying Cramer’s *V* statistics indicated that decedent sex and age variation have a limited relationship (i.e., small effect) with agency of origin, while race and/or ethnicity variation appears to be more strongly related to agency of origin, with a medium effect.

**Table 4:** Results of the Chi-square tests for comparing inter-agency demographic variation and identification rates. Shaded cells indicate statistical significance of test for the adjusted *p* values.

Test	n	df	Likelihood Ratio Chi-Square	Adjusted *p*	Cramer’s *V* 95% CI	*R* ^2^	Cramer’s *V* Effect Size
**Agency x decedent sex**	1196	2	17.72	0.00024	0.1194 (0.066–0.178)	0.012	small
**Agency x decedent age**	1132	8	88.77	0.00024	0.194 (0.164–0.237)	0.029	small
Agency x decedent race*	965	6	285.98	0.00024	0.388 (0.352–0.430)	0.126	medium
**Agency x identification status**	1253	2	24.54	0.00024	0.140 (0.088–0.196)	0.014	small

We compared the identification rates among the three agency samples (Table 3) and found that identification rates ranged from 41% to 57%, with NYC identification rates being the greatest. The Chi-Square test for frequency differences in identification rates among the three agencies was a significant (Table 4), but small effect. When pooled, the identification rate for the sample was 47%.

### Aim 1: For forensic anthropology cases, do investigation trends (e.g. identification success rate; duration of an investigation) differ across demographic backgrounds

#### Pooled sample univariate analyses

**Fig 1:**
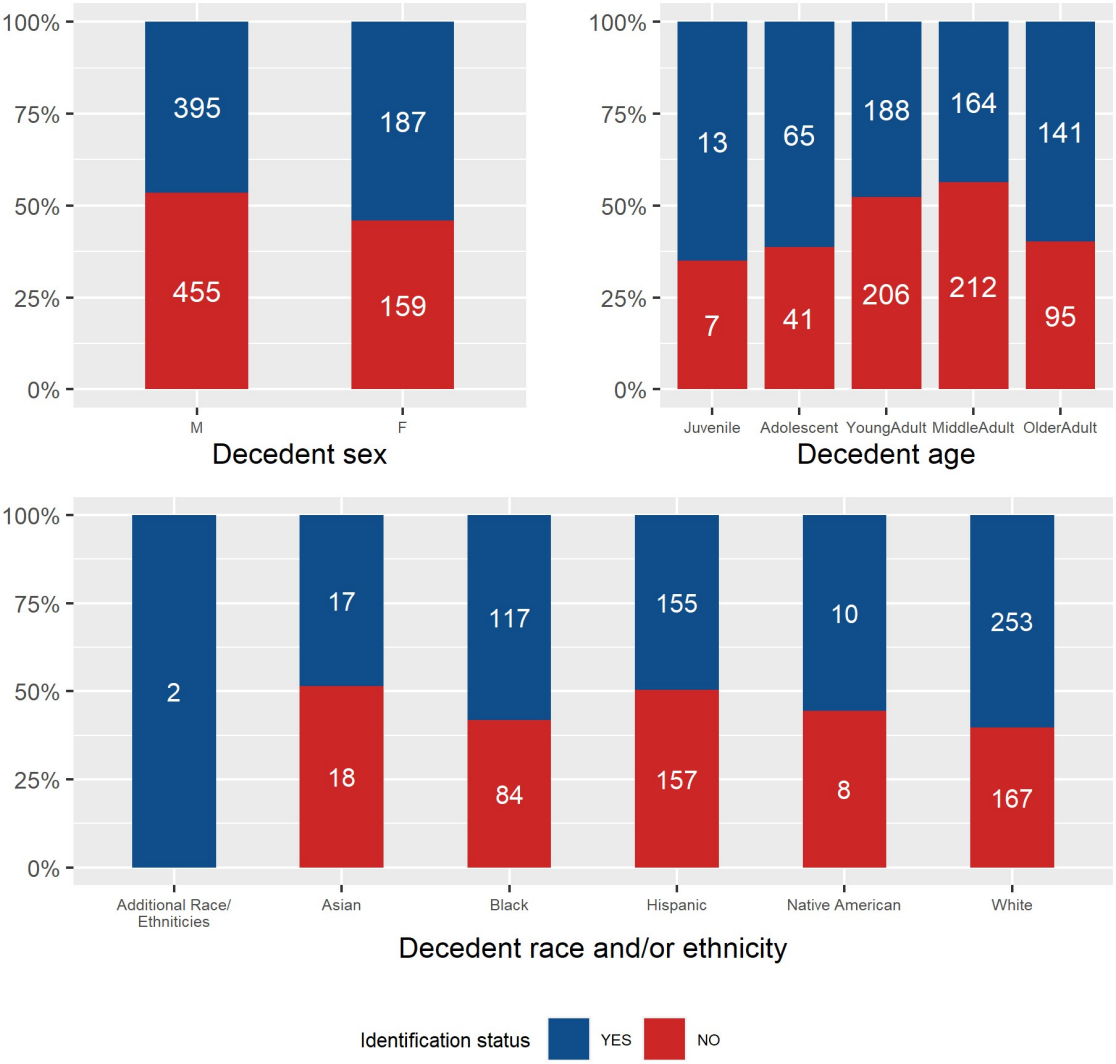
Pooled sample identification rates (0–100%) per decedent demographic data.

Using the pooled sample, we then explored the relationship between identification status (either identified or not identified at the time this data was collected) and its associated decedent demographic data (sex, age, and race and/or ethnicity). Cases where inferred decedent data for sex, age, or race and/or ethnicity were lacking were excluded from each analysis. The pooled samples’ Chi-square results for examining identification rate trends across decedent demographics are presented in Table 5, and the identification frequencies associated with demographic data are presented in Fig 1. Chi-Square tests and Cramer’s *V* were employed to identify any statistically significant and notable trends. The pooled sample’s results suggest that identification frequencies statistically differed with decedent age and sex, but not decedent race and/or ethnicity. The Cramer’s *V* measure of effects for age and sex effects on identification status are small and negligible, respectively, thus indicating limited differences among the age and sex subsamples’ identification rates. For sex, females (54% identification rate) were more frequently identified than males (47% identification rate). Fig 1 also highlights that the main frequency differences for age indicate that juvenile, adolescent, and older adult subsamples have higher rates of identification when compared with middle and young adult subsamples.

**Table 5:** Tests of identification rate variation and decedent demographic data. Pooled and agency-specific tests performed. Shaded cells indicate significance of adjusted *p*-value.

Test	n	df	Likelihood ratio (Or Fishers)	Adjusted *p*	Cramer’s *V* 95% CI	*R* ^2^	Cramer’s *V* Effect Size
**ID status x decedent sex (pooled)**	1196	1	5.65	0.0255	0.069 (0.012–0.125)	0.003	negligible
**ID status x decedent age (pooled)**	1132	4	23.21	0.00024	0.143 (0.097–0.211)	0.015	small
**ID status x decedent race (pooled)**	996	5	9.338	0.105	0.095 (0.054–0.171)	0.007	n/a
**~Agency specific tests below~**							
*ID status x decedent race (FBI sample)	414	4	6.17	0.189	0.119 (0.068–0.223)	0.011	n/a
**ID status x decedent race (NYC sample)**	287	3	4.57	0.24	0.122 (0.055–0.251)	0.013	n/a
**ID status x decedent race (UNT sample)**	275	2	31.48	0.001	0.330 (0.227–0.433)	0.083	medium
**ID status x decedent sex (FBI)**	510	1	2.854	0.127	0.075 (0.007–0.161)	0.004	n/a
**ID status x decedent sex (NYC)**	357	1	0.663	0.415	0.041 (0.002–0.144)	0.001	n/a
**ID status x decedent sex (UNT)**	327	1	3.73	0.088	0.108 (0.013–0.214)	0.008	n/a
**ID status x decedent age (FBI)**	476	3	11.4	0.0343	0.155 (0.082–0.252)	0.018	small
**ID status x decedent age (NYC)**	332	4	9.85	0.0602	0.168 (0.095–0.293)		n/a
**ID status x decedent age (UNT)**	316	3	9.97	0.063	0.175 (0.100–0.294)	0.023	n/a

#### Agency-specific analyses

**Fig 2:**
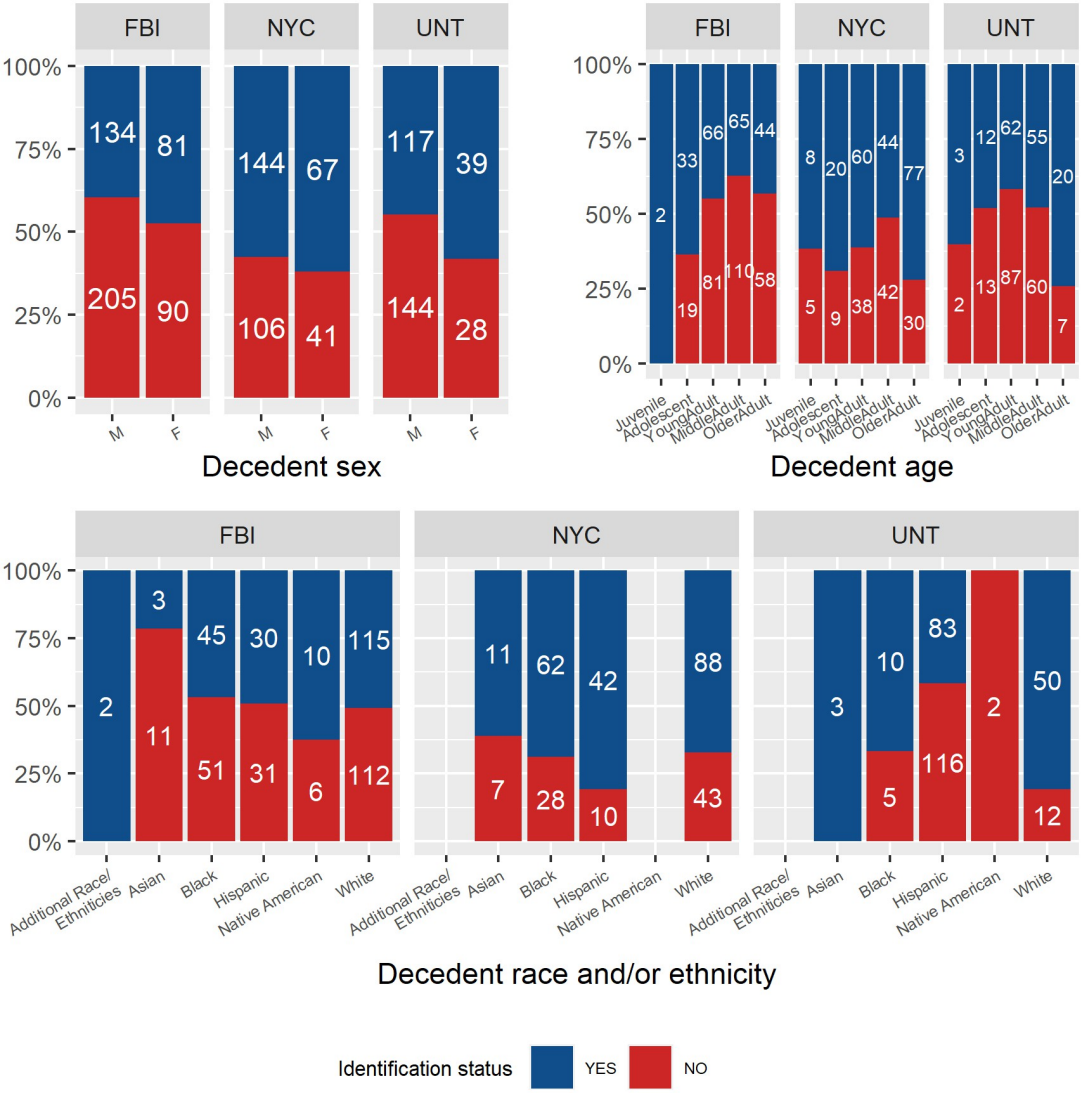
Agency-specific identification rates (0–100%) per decedent demographic data.

Because pooling the sample could be masking agency specific trends, we again examined the relationship between identification rates and decedent demographic data (sex, age, and race and/or ethnicity) per agency sample. The identification rates per agency are reported in Fig 2, and the Chi-square results are presented in Table 5. We found that none of the three agencies yielded significantly different identification rates for males and females. For decedent age, we found that only the FBI sample’s identification rates significantly differed among age subsamples. While there were no significant differences in identification rates among age subsamples for UNT and NYC, their trends indicate agreement among the agencies in regard to identification rates, with Older Adult subsample having the greatest identification rates (74% and 72%, respectively). For the FBI and NYC samples, the Adolescent case subsamples had some of the highest identification rates (64% and 69%, respectively). The Middle Adult subsample exhibited the lowest identification rate for the FBI (47%) and NYC (51%) samples, and the second lowest for the UNT (49%) sample.

When examining the relationship of decedent race and/or ethnicity with case identification rates, agency-specific datasets did not adequately represent all available subsamples used in the pooled sample. For NYC, Native American decedents were excluded, and for UNT Native American and Asian decedents were excluded. The FBI dataset had no groups excluded. The only agency with statistically significant differences in identification rates across race and/or ethnicity was the UNT sample (Table 5), with a medium effect size. For the three groups examined (Black, Hispanic, and White), White decedents’ identification rate (81%) was twice that of Hispanic decedents’ (42%), while the Black decedent identification rate was intermediate (67%). In contrast, NYC Hispanic decedents present the greatest identification rate (81%) compared to Asian (61%), Black (69%) and White (66%) decedent identification rates. The FBI analysis had highly comparable identification rates for Black, Hispanic and White decedents (47–51%), while Native American and Asian identification rates were the greatest (63%) and lowest (21%), respectively. Given that the only agency with significantly different identification rates among race and ethnicities is the UNT sample, this suggests that the statistical significance of this same test when using the pooled sample is likely driven by the differences found within UNT, and not a nation-wide trend of lower identification rates for Hispanic decedents. It should be noted that the small samples for decedents—namely, FBI Asian (n = 14), FBI Native American (n = 16), and UNT Black (n = 15)—may not be representative of United States identification trends at large for these subsamples, and more data will be needed to better gauge the identification trends.

#### Pooled multivariate analyses

**Fig 3:**
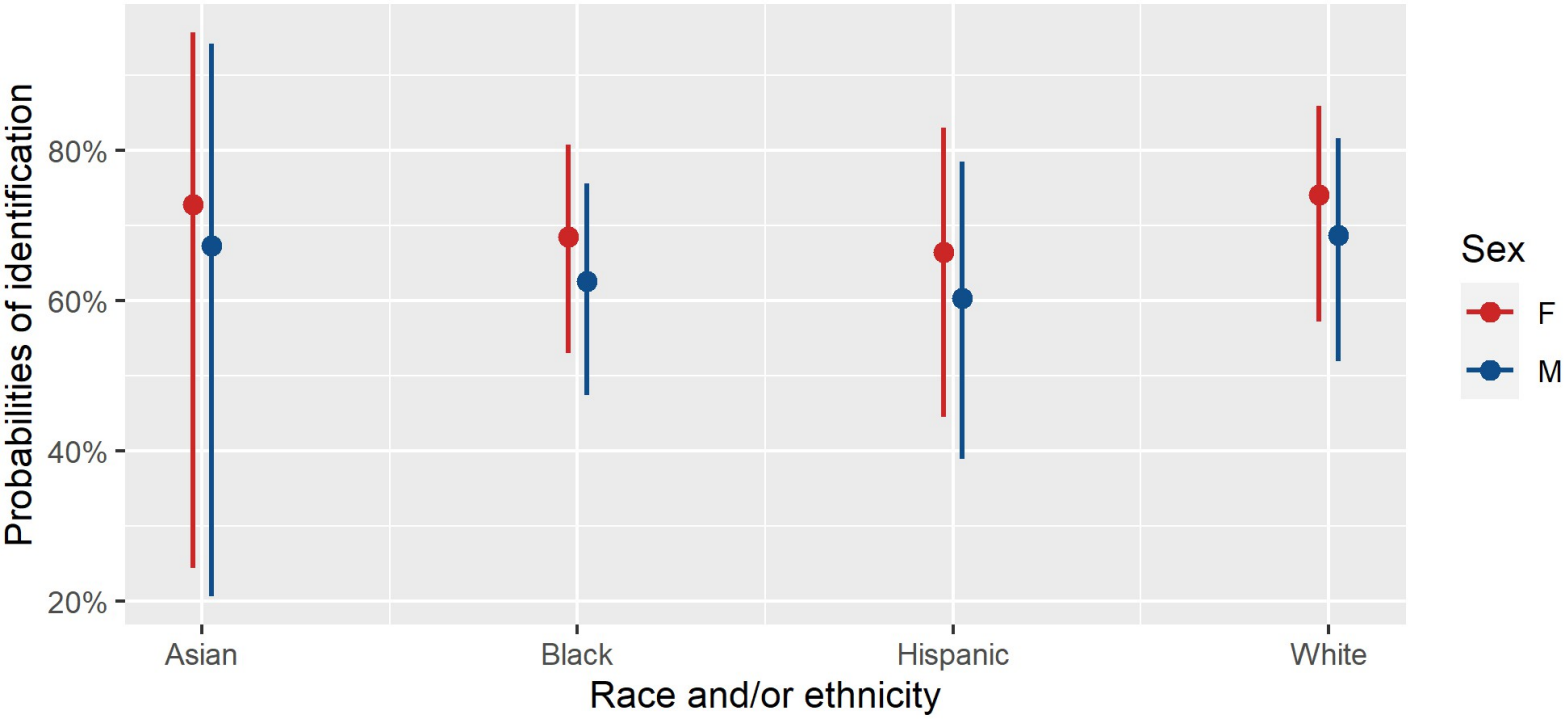
Estimated probabilities of identification for the combination of demographic factors (sex and race and/or ethnicity). Note the overlaps among subsamples suggest the effects of demographic factors on identification probabilities are small.

While the univariate tests indicate trends for the independent factors of decedent sex, age, and race and/or ethnicity, it is also important to ensure that we take into account the multivariate nature of demographic data, as individuals simultaneously belonging to a specific combination of sex, age, and race and/or ethnicity for any given case, as well as considering agency of origin. This approach most strongly corresponds with the reality of casework, in that a decedent’s sex, age, and race and/or ethnicity would typically be simultaneously considered in a case and investigated within a particular agency. Thus, we employ a multivariate approach to more realistically infer the relationship between identification rates and decedent demographics. The multiple logistic regression model indicated the overall effects of decedent sex, age, and race and/or ethnicity were not significant, after agency effects were taken into account (Table 6). The results of the logistic regression model also revealed the direction of identification rate differences while taking all demographic factors into account, and the results were consistent with the univariate findings. All other factors equal, females are more likely to be identified than males, Middle Adults are less likely to be identified than other age groups, and White and Asian decedents are more likely to be identified than other race and/or ethnicity. However, the amount of overlap in conditional probabilities among different categories for each individual predictor variable (sex, age, and race and/or ethnicity) again suggest the differences in identification rates among the subsamples are almost nonexistent for sex and race and/or ethnicity, and small for age, primarily driven by the Middle Adult group’s lower identification rates deviating from all other age groups. The model also allows us to explore the combination of factors such as sex and race and/or ethnicity as they relate to identification status (Fig 3). Regardless of age, White females and Asian females have slightly higher probabilities of identification compared to other subsamples. However, the amount of overlap among subsamples is big and suggests that the effects of demographic factors to identification status, if any, are small.

**Table 6:** Odds ratios of identification status estimated from the multivariate mixed effect logistic regression model. The reference categories (odds ratio = 1) are female, adolescent, and White.

Predictors	Odds Ratio	CI	*p*
**Sex**			0.1336
**Male**	0.77	0.55–1.05	0.101
**Age**			0.2641
**Young Adult**	0.68	0.38–1.23	0.204
**Middle Adult**	0.53	0.27–1.04	0.061
**Older Adult**	1.01	0.43–2.37	0.978
**Race and/or ethnicity**			0.8694
**Asian**	0.97	0.21–4.47	0.973
**Black**	0.78	0.44–1.35	0.37
**Hispanic**	0.71	0.21–4.47	0.572

#### Investigative duration trends

To explore whether duration of an investigation differed across decedent demographics, we examined the relationship between investigation duration and decedent sex, age, or race and/or ethnicity using the UNT identified case sample (Table 7). Employing Wilcoxon Rank Sum/Kruskal Wallis tests, we then tested for differences in the investigation duration distribution across decedent sex, age, and race and/or ethnicity. Subsamples analyzed for these analyses were limited to those with n ≥ 20, resulting in the following groups per decedent demographic component for the identified UNT case sample: decedent sex (Male and Female), decedent age (Young Adult, Middle Adult, Older Adult), and decedent race and/or ethnicity (Hispanic and White). Ninety-eight percent of the cases used in this analysis were DNA identifications, meaning that when holding identification modality constant, we can explore the effects of demographics on investigation duration. It has been hypothesized [1, 34] that anthropological estimations of sex, age, or ancestry could influence investigations such that some demographics’ cases are prioritized over others resulting in lengthier investigation times for deprioritized groups. Therefore, in Table 7 we also report the percent of cases with corresponding biological profile estimation information available in the UNT samples analyzed for investigation duration trends. The vast majority of cases had their biological profile information available at some point in the investigation, and therefore it can be assumed that if this information was to bias the investigation in some way, we would see that emerge as differences in the investigation duration trends. The median investigative duration for all seven groups was one year, and the distributions of the investigative durations did not significantly differ among the tested sexes nor among the tested age groups. Regarding the race and ethnicity, recall from the Methods section that for the comparisons of Hispanic and White decedent investigation durations we employed for two case year cutoffs, 2012 and 2014. The 2012 analysis resulted in significantly different investigation duration distributions (but identical medians at 1 year), primarily in the upper quantiles (75%-100%) of the distribution, with Hispanic decedents having greater investigative durations than White decedents (2–5 years *versus* 1–3 years). In contrast, the 2014 analysis did not find significant differences in investigation durations for White and Hispanic decedents.

**Table 7:** Wilcoxon/Kruskal Wallis Tests for investigation duration differences among decedent demographics. Shaded cells indicate significance of adjusted *p*-value.

Decedent Demographic	n	Test Statistic	Adjusted *p*	% of cases with corresponding biological profile estimation
**Sex**	145	3.94	0.0883	93%
**Age**	126	1.42	0.548	100%
**Race (cases 2012-Present)**	108	6.891	0.044	82%
**Race (cases 2014-Present)**	87	2.046	0.191	92%

### Aim 2. Is the decedent information included in forensic anthropology reports (e.g., estimations of decedent ancestry, sex, and age) related to the (de)prioritization of particular individuals over others in regard to identification efforts

Decedent demographic trends are analyzed similar to Aim 1, but with the dataset comprised of only forensic anthropology *estimated* demographic data. In this way, we are able to compare how forensic anthropologists’ estimated demographic information about a decedent are related to investigative trends. The identification trends for *only* estimated decedent sex, age, and ancestry were generally comparable to those found in Aim 1 using *known* and *estimated* decedent data, with a few exceptions highlighting greater demographic discrepancies. In particular, for the pooled agencies analyses, all three demographic factors (estimated sex, age, and ancestry) yielded significant differences in identification success rates (S1 Table in S1 File), although the Cramer’s *V* effect sizes were negligible for estimated sex and small for estimated age and ancestry. Decedents estimated as White and Black had the greatest investigative success with 54% and 53% of cases identified, respectively. Clustered with lower investigation success rates (39%-41%) are decedents estimated to be Asian, Hispanic, or multiple labels. Agency-specific analyses of estimated demographic data and identification rates indicated no significant relationships for the FBI sample. For NYC, there was a significant relationship (small effect) between identification rate and estimated ancestry such that decedents identified as Multiple labels were less frequently identified. For UNT, both estimated age (small effect) and ancestry (medium effect) were significantly related to identification rate, with Young Adult decedents as well as Hispanic decedents less frequently identified than other groups. Agency-specific investigation duration trend analyses indicated no significant differences in time to identification for the included UNT sample (S2 Table in S1 File).

The multivariate results, which allow for the composite demographic estimates to be observed are somewhat different from the results of univariate analyses, showing the overall effects of estimated sex, age, and ancestry were not significant, after agency effects were taken into account (S3 Table in S1 File). This is consistent with the multivariate results for Aim 1. For the combination of estimated sex and ancestry and how they relate to identification status (S1 Fig in S1 File), the 95% confidence intervals for the probabilities of identification all overlap. The wide identification probability CIs associated with decedents estimated to be Asian females and females with multiple ancestry labels are likely associated with their small sample sizes, and thus the lower probabilities of identification need further investigation with more robust samples. For the other six group, White and Black males and females have substantial overlap in their 95% CIs for identification probability, while Hispanic males appear to be the lowest and have limited overlap with the White males. This trend is consistent with odds ratio analyses (S3 Table in S1 File), where the only statistically significant odd ratios was White decedents being twice as likely to be identified as Hispanic decedents.

### Aim 3. Does providing information related to the decedent’s age, sex, and race impact the odds of an identification?

**Fig 4:**
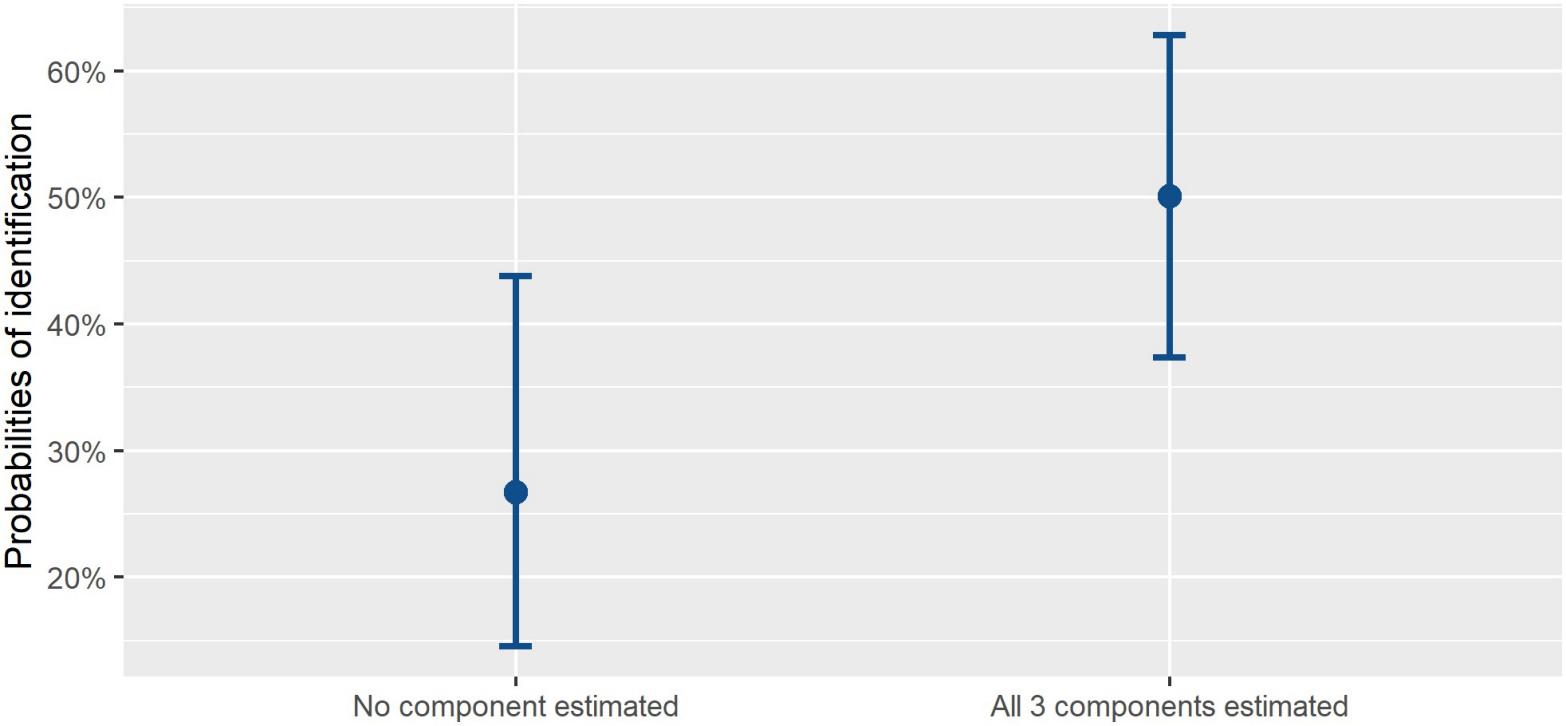
Comparison of the probabilities of identification for cases when no biological profile components are estimated *versus* when sex, age and ancestry are all estimated. An increase in identification probability is evident when all biological profile components are estimated (26.7% probability of identification with no biological profile component estimated vs. 50.1% probability of identification with all biological profile components estimated).

We examined whether biological profile estimates provided by the forensic anthropologist (e.g., sex, age, or ancestry) have a relationship with identification rates. We performed a mixed effects logistic regression to infer whether identification rates related to whether or not forensic anthropology decedent demographic estimates were provided (e.g. “provided” or “not provided”), taking case agency into account. The odds ratios for the presence of biological profile components are as follows: 2.16 (95% confidence interval: 0.72–6.44, p > 0.05) for sex, 1.21 (0.47–3.15, p > 0.05) for age, and 1.05 (0.47–2.34, p > 0.05) for ancestry) indicated that estimations of each biological profile component only had a limited relationship with the probability of identification. When all three components were estimated (compared to when no component was estimated), the probability of identification increased twofold (Fig 4).

To better understand which agencies are contributing to the observed differences in identification rates in the multivariate models, we examined the relationship between identification status and the presence or absence of forensic anthropology estimates of sex, age or ancestry for the agency-specific samples using univariate tests (Table 8). We found that NYC identification rates significantly improved when sex, age, or ancestry estimations were provided by 40%, 27% and 25%, respectively, compared to when these estimations were not provided, but all were considered small effects. In contrast, the FBI and UNT presented no significant differences in identification rates related to the presence or absence of sex, age, or ancestry estimations.

**Table 8:** Testing for the effect of reporting vs. not reporting bio profile information (sex, race, or both sex and race) on identification rate. The "n/a" status for the UNT age test is because age was estimated in all cases and thus the test could not be performed. Shaded cells indicate statistical significance of test for the adjusted *p* values.

Test	n	df	likelihood ratio	Adjusted *p*	Cramer’s *V*; 95% CI	*R* ^2^	Cramer’s *V* Effect Size
**Sex estimated (Y or N) x ID status**	1253	1	8.21	0.0072	0.080 (0.027–0.133)	0.005	negligible
**FBI**	524	1	1.95	0.198	0.062 (0.005–0.148)	0.0028	n/a
**NYC**	367	1	23.274	0.0007	0.249 (0.154–0.345)	0.046	small
**UNT**	361	1	3.122	0.110	0.092 (0.008–0.193)	0.006	n/a
**Ancestry estimated (Y or N) x ID status**	1253	1	0.574	0.449	0.021 (0.001–0.080)	0.0003	n/a
**FBI**	524	1	3.761	0.0917	0.085 (0.008–0.173)	0.0053	n/a
**NYC**	368	1	14.289	0.0007	0.198 (0.091–0.296)	0.029	small
**UNT**	360	1	0	0.99	0.001 (0.001–0.111)	0	n/a
**Age estimated x ID Status**	1248	1	10.99	0.0018	0.092 (0.039–0.141)	0.0064	negligible
**FBI**	519	1	4.643	0.072	0.093 (0.016–0.172)	0.0066	n/a
**NYC**	368	1	6.19	0.0231	0.130 (0.028–0.221)	0.012	small
**UNT**	n/a	n/a	n/a	n/a	n/a	n/a	n/a

We performed a follow-up analysis which explored whether having an ancestry estimation available (two levels: ancestry estimation provided; ancestry estimation not provided) differentially impacted the identification outcome across decedent race and/or ethnicity. This analysis was performed to explore whether trends exist that suggest cases are (de)prioritized by the ancestry information that the forensic anthropologist provides. For example, if unidentified persons investigations are biased towards prioritizing cases of White decedents, then we would expect to see a greater proportion of White decedents in the sample of positively identified cases *with* ancestry estimations, compared to the positively identified case sample *without* ancestry estimations. Therefore, we analyzed whether the proportion of race and/or ethnicity groups differed between identified individuals with ancestry estimations *versus* identified individuals without ancestry estimations. In this scenario, the subsample of identified case *without* an ancestry estimation is considered the control sample, as there is no information on the decedent’s possible race and/or ethnicity to potentially bias the investigation, and thus the proportions of race and/or ethnicities in this subsample will be considered the control. Alternatively, the identified case sample *with* ancestry estimations can be considered the biased case subsample, as there is information on the decedent’s possible race and/or ethnicity, which has the potential to bias the investigation.

**Fig 5:**
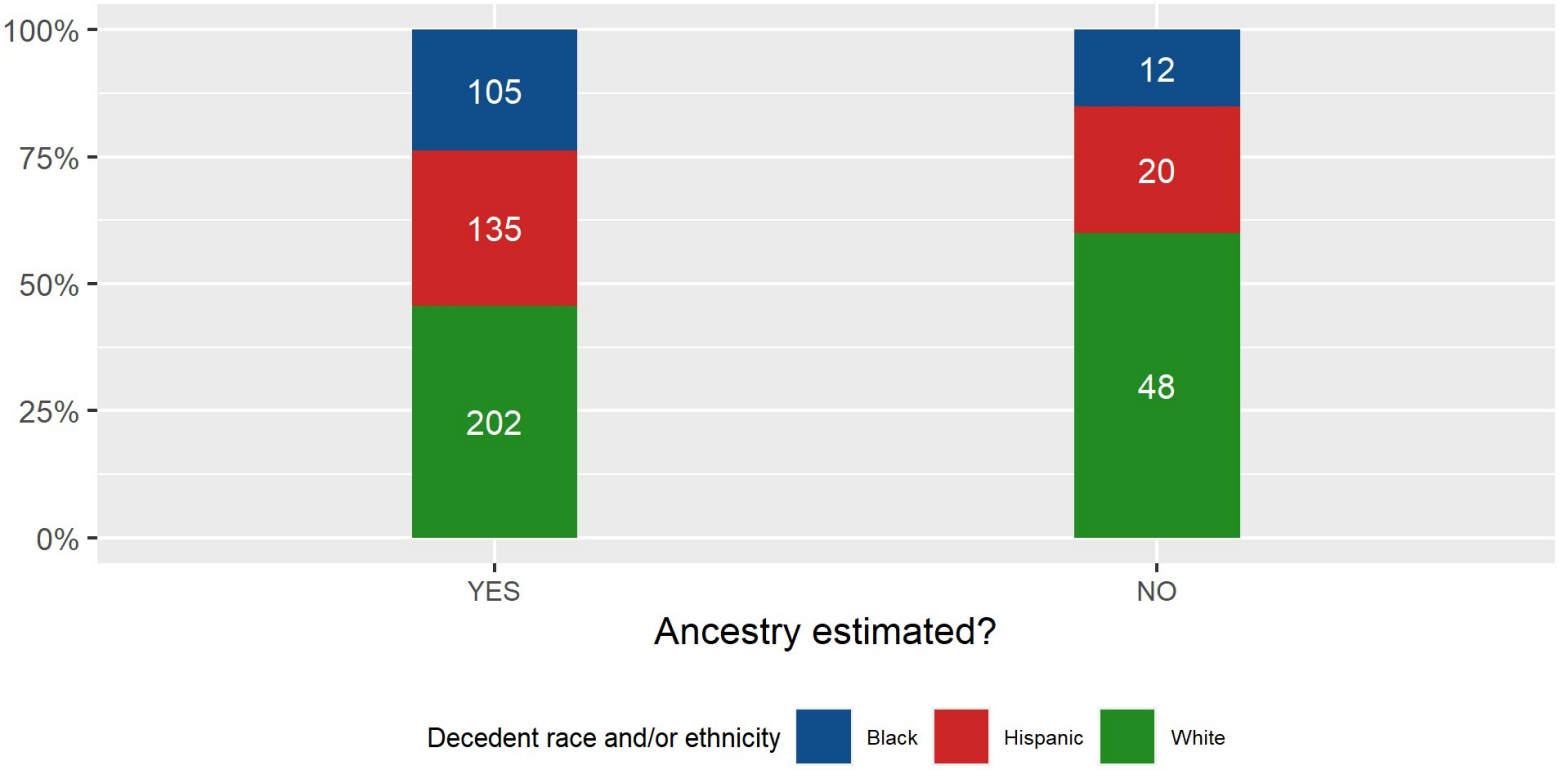
Identified case sample, comparing the proportion of decedent race and ethnicity subsamples for when ancestry was and was not estimated.

For this analysis, identified individuals representing three decedent races and/or ethnicities (those with substantial sample sizes being Black, White, and Hispanic decedents) from all agencies were used. We found that race and/or ethnicity proportions of identified cases (Fig 5) were not significantly related to whether or not an ancestry estimation was reported (*N* = 522, adjusted *p* = 0.066; Cramer’s *V* = 0.106). While not significant, it is interesting to note the trends observed, with both Black and Hispanic decedents exhibiting higher proportions (9% and 6% higher, respectively) in the identified case subsample when ancestry *was* estimated, while White decedents had a concomitant decrease (14% decrease). Similar to the trends of the pooled sample, the agency specific analyses showed an increase in the identification of Black decedents when ancestry is reported. However, only UNT exhibits significant differences between Black, Hispanic and White decedent proportions for identified individuals with *versus* without an ancestry estimation (Fisher’s Exact Test, *N* = 143, *p* = 0.028 Cramer’s *V* = 0.225). For UNT, the identification of Hispanic individuals almost doubles (to 63%) when ancestry information *is* reported, compared with White decedents whose frequency is almost halved (30%).

Similar analyses could not be performed for decedent sex or age because there are only a limited number of identified decedents without forensic anthropology estimates of age or sex, and thus inadequate comparative data of identified decedents with or without this biological profile information. An unreported biological profile estimation may be due to several factors, including but not limited to incomplete and/or damaged skeletal material, methodological outcomes with weak probabilities, or contradicting outcomes from various methods.

## Discussion

This study examines the broader context of identification trends for forensic anthropology casework, as it relates both to decedent demographics and the information provided by the forensic anthropologist (the biological profile estimations). We examined these trends for both pooled and agency-specific samples because agency contexts (e.g., jurisdiction of cases, decedent demographics of casework) significantly differ among the three datasets included in the present study.

### Do identification trends vary across decedent demographics?

In Aim 1, we examined the relationship between identification rates and decedent demographics (known and estimated) using both univariate and multivariate approaches. The multivariate logistic regression offered the most comprehensive analysis in that it allowed for the *simultaneous* consideration of sex, age, and race and/or ethnicity of each decedent as well as the agency effects. The model indicated the overall effects of decedent sex, age, and race and/or ethnicity on identification rate were not significant (Table 6). These findings corroborate the univariate analyses of the pooled sample, which indicated that while both decedent age and sex significantly related to identification rate, their overall effects on identification rate were small or negligible, and appear to become insignificant when considered in tandem with other relevant factors in the multiple logistic regression. When comparing to the agency-specific univariate tests where the only significant finding was a relationship between identification rate and decedent race and/or ethnicity for UNT, this factor did not remain significant in the multiple logistic regression when agency differences were taken into account. These Aim 1 analyses suggest that decedent sex, age, and race and/or ethnicity have a limited (univariate analyses) to negligible (multivariate analysis), relationship with identification rates for the study samples.

Beyond identification rate, duration of the investigation for resolved cases was examined in Aim 1. The significant increase in investigation duration for UNT cases from 2012 to present for Hispanic decedents compared to White decedents compared to the lack of significantly different investigation durations for Hispanic and White decedents by 2014 may be explained by the changes of undocumented migration patterns around this time. Deaths of undocumented migrants in Texas were substantially increasing around this time, surpassing Arizona’s numbers in 2012 [63]. The influx of undocumented migrant deaths could have impacted UNT’s caseload, with needed investigative adjustments to accommodate the rise in this decedent demographic. By 2014 (through 2021), we found that the investigation durations were no longer elevated for Hispanic decedents (compared to White decedents), which could correspond with the sustained uptake of investigative strategies by the agencies served by UNT and the UNT Forensic Anthropology Unit itself. Interestingly, even though UNT’s Hispanic and White decedents exhibited the most significant difference in *identification rates*, in more recent years (2014 to present) when investigations do yield an identification, Hispanic and White decedents do not appear to significantly differ in the amount of time it takes to make those identifications. Furthermore, comparable investigation durations across subsamples cannot be explained by cases that come in with tentative identifications nor the modality of the identification, as those were controlled for. For future studies, examining this variable using a finer scale, such as months instead of years, and with a larger data set from more agencies would be ideal to corroborate the trends found with the available UNT data.

### Do identification trends vary across estimated decedent demographics?

Aim 2 performed similar analyses to Aim 1, but with a sample whose decedent information was entirely based on the forensic anthropology biological profile estimates. In this way, Aim 2 explicitly examined the relationship of identification trends with the *estimated* decedent demographics data, which are what investigators would have been privy to during their investigations. Recall that it is this estimated demographic information that Bethard and DiGangi [1] proposed could influence the way in which an investigator (de)prioritizes one case over another. In general Aim 2’s univariate trends were similar to the Aim 1, with small to negligible effects for most estimated decedent demographics, and with a medium effect for estimated ancestry. Aim 2’s multivariate analysis of identification rates across various estimated decedent demographics indicated that only ancestry was influential, but the only statistically significant odds ratio for being identified (S3 Table, S1 Fig in S1 File) was for decedents estimated as Hispanic *versus* White at 0.44. This finding is consistent with the univariate results for Aims 1 and 2 where White decedents were identified at twice the rate as Hispanic decedents for UNT cases. While not significant, the FBI’s analysis also indicated that Hispanic decedents had the lowest rate of identification among those analyzed. However, agency variation is important to acknowledge, as Hispanic decedents had the highest identification rates in NYC at 75%, which was the greatest identification rate for *any* decedent ancestry, race, and/ethnicity examined. Furthermore, similar to Aim 1, even within an agency with a low rate of identification of Hispanic cases like UNT, Aim 2’s analysis of investigation duration did not result in statistically significant differences for the three compared groups (S2 Table in S1 File). Collectively, Aim 2 exhibited more statistically significant differences in identification rates than Aim 1, suggesting that the particular information provided by forensic anthropologists has a relationship with identification outcomes.

### Does the availability of biological profile estimations relate to identification trends in forensic anthropology cases?

We also addressed the question of identification discrepancies when biological profile estimates are provided to investigators. Through univariate and multivariate testing, we found that identification rates appear to have a significant but limited relationship with the availability (e.g. presence or absence of this information) of biological profile estimations. Trends from the pooled sample’s multiple logistic regression indicate that decedents with all three biological profile components present in a case report have a greater probability (50%) of being identified compared to decedents who have none of these biological profile components estimated (27%). Agency-specific univariate analyses yielded further insight (Table 8), highlighting that NYC was the only agency where the presence of sex, age, and ancestry estimations each independently significantly improved identification rates by 40%, 27% and 25%, respectively.

The relationship between forensic anthropological analyses/involvement with a case and identification rates can also be considered within the nationwide context of identification rates. The pooled FBI, NYC, and UNT identification rate (47%) is almost double the rate of identifications of unknown individuals uploaded to NamUs. Out of the 18,740 unidentified individuals uploaded, approximately 27% have since been identified (data accessed on 7/28/2021). Interestingly, cases of unidentified individuals uploaded to NamUs also include individuals with a tentative name upon the initiation of the investigation which presumably have a better chance of being identified than those without a lead. However, individuals with a tentative name were excluded from the present study, which indicates that the identification rates reported here are likely an underrepresentation of the overall identification rates for U.S. forensic anthropology cases.

One key difference between NamUs and the present study is that NamUs includes all cases of unidentified individuals, while the present study only includes unidentified individuals with a forensic anthropological biological profile (though not necessarily complete). Any discrepancy in overall identification rates between NamUs and the present study could also be tangentially related to the agencies’ represented in the two datasets—specifically, that agencies that hire in-house forensic anthropologists are inherently larger with more local resources (e.g., NYC cases and some of the FBI and UNT cases) to put towards investigations in general, which may indicate that these agencies have a better chance of identifying individuals. Additionally, those agencies using external forensic anthropologists (such as the agencies represented in the FBI and the UNT samples) could also represent smaller but actively engaged agencies with a better understanding of how to leverage the available resources at the local and national level (e.g., UNT and some of the FBI cases), which may in turn increase their odds of identification. Finally, the discrepancy in identification rates between NamUs and the present study could be *directly* related to the role of the forensic anthropologist, in that the estimation of a biological profile may increase the chance of identification and/or the kind of general engagement forensic anthropologists have with casework improves the odds of an identification. Because there are potentially confounding factors in an investigation that could result in a spurious association of the availability of forensic anthropology estimates and the chance of identifications (e.g., poorly preserved/recovered skeletal remains), we cannot infer a causal relationship from these results. While the effects size for the NYC univariate tests were small, the significant results of the multivariate analysis (which took agency differences into account) and the consistency with broader national trends suggest improvements to identifications when the biological profile is provided and that more research would be useful to infer whether this positive impact (though small effect) of forensic anthropology reporting holds beyond the present study sample.

Lastly, we addressed the specific question of identification discrepancies when ancestry estimates are provided to investigators by examining whether identification trends vary with the availability of reported biological profile estimates. After the multivariate finding that the availability of biological profile information has a significant relationship with the odds of an identification, it is important to understand whether biological profile information has the same relationship with identifications for different decedent demographic groups. Namely, we examined the postulation that biological profile estimations of ancestry can inadvertently prioritize identifications of one decedent over another, specifically cases of White decedents [1, 34]. In this context, any case where ancestry was estimated has the potential to bias investigators in the direction of prioritizing White individuals and deprioritizing all others, which would result in a *larger* proportion of White individuals in the *identified* case subsample with ancestry estimations, and a smaller proportion of White individuals in the identified case subsample without ancestry estimations. Any identified individual without an ancestry estimation would have had no information that could bias the investigation toward prioritizing decedents of a certain race over others. Using the identified case sample, we found no differences in decedent race and/or ethnicity proportions when ancestry was and was not estimated (Fig 5), suggesting limited evidence that prioritization of certain groups over others is occurring for the present study sample for this analysis.

Interestingly, in the agency-specific analyses, UNT exhibited a significant trend that is opposite of the exhibited identification rate trends of White decedents twice as frequently identified as Hispanic decedents. When ancestry was estimated, the proportion of Black and Hispanic individuals *increased*, while the proportion of White decedents *decreased* when compared to unidentified remains cases where ancestry was *not* estimated. This trend suggests a clear relationship between decedent race and/or ethnicity, identification, and available ancestry estimations. While prioritization of Black and Hispanic individuals in investigations is one way to interpret these findings, an alternative explanation is that estimations of ancestry improve the odds of an identification for Black and Hispanics decedents more so than for White decedents. This aligns with previous work [64] that indicates the evidentiary value of an ancestry estimation towards an identification is largely based on the racial diversity of the pool of missing persons. Given that UNT cases originate from all over the United States, the nationwide pool of missing persons is largely represented by White individuals (59%), making an ancestry estimation related to White the least unique/lowest evidentiary value for contributing to an identification. Alternatively, ancestry estimations related to Black and Hispanic decedents would have greater evidentiary value, as Black and Hispanic individuals comprise only 16% and 11% of the missing person pool, respectively (NamUs [65]). However, if evidentiary value was the main factor contributing to the observed differences, then we should have found a similar trend in the FBI as we did with UNT, given that the FBI sample also consists of nation-wide cases. Other factors, such as the lack of missing persons report being filed would also impede the ability for biological profile estimations to assist in an identification. Further study of these trends and potential factors contributing to them will help to elucidate the relationship among decedent race and/or ethnicity, the availability of ancestry estimations, and identifications in casework.

### Providing context: Factors influencing investigative trends

In this study, the majority of investigative trends related to decedent demographics are negligible to small effects. Notably, decedent ancestry, race, and/or ethnicity exhibited medium effects for Aims 1 and 2 in the univariate analysis for the UNT sample. These findings provide evidence consistent with our hypotheses for Aims 1 and 2, in that decedent demographics are related to identification rates. One interpretation of these findings is to conclude that (de)prioritization of cases based on the decedent demographic is what is driving these results. However, the results were inconsistent across univariate and multivariate tests, as well as at times contradicting when comparing various measures of (de)prioritization we employed. For example, while identification *rates* appear to be significantly related to decedent demographics, identification *duration* did not always demonstrate similar results. Furthermore, Aim 3 found no differences in decedent race and/or ethnicity proportions subsamples when ancestry was and was not estimated (Fig 5), suggesting limited evidence for prioritization. Collectively the inconsistency in the results are unsurprising, given that whether or not active (de)prioritization is occurring by investigators, that is just one of myriad factors which contribute to each cases’ probability of being identified. In the following paragraph, we discuss some of these factors and how they offer additional considerations beyond only (de)prioritization to contribute to the observed trends in our study.

When considering decedent sex, females exhibited significantly greater identification rates compared to males, although the overall difference is less than 10% with a negligible effect per Cramer’s *V*. There are several contextual factors that could play a role in the increased identifications of females, such as females being reported missing more often than males, and/or overall fewer missing and unidentified females such that there is inherently a smaller pool of potential matches in the investigation of unidentified females. National statistics on missing and unidentified persons reflect these trends of greater reporting yet lower prevalence among the unidentified, with females only comprising 19% of the unidentified persons in NamUs, yet 39% of the reported missing persons (NamUs [65]).

Age groups were found to be significantly related to identification rates for the pooled sample, with Juveniles, Adolescents and Older Adults having better identification rates than Middle and Young Adults. However, this same trend was not found to be significant in the multiple logistic regression, nor the agency-specific univariate tests, with the exception of the FBI sample which showed only a small effect driven by the Adolescent group outperforming (greater identification rates) Young, Middle, and Older adult groups. It is possible that law enforcement may not always take missing persons reports for missing young and middle-aged adults because they could be viewed as going missing voluntarily, thus contributing to these cases remaining unidentified. In addition, juvenile, adolescent and older adult age subsamples are intentionally included in various societal systems of surveillance that work simultaneously to aid in identifications and may in part explain the improved identification rates compared to other age subsamples. According to a 2021 National Center for Missing and Exploited Children (NCMEC) report [66] in which identifications of deceased children were analyzed, individuals aged 11–20 represent the largest percentage of deceased and identified children (65%)in the study. Thus, among child/adolescent deaths, this age range is both the most commonly deceased and the most commonly identified. Children in this age range have various forms of surveillance as part of their daily lives (e.g., school, parents, caregivers), and as such their disappearance is commonly noted within this system. According to the NCMEC report, the majority of deceased children were recovered in the same city or state where they went missing, and identifications primarily stemmed from law enforcement tips. This “closed system” empowers these local systems of surveillance and potentially increases identifications. In addition, cases of child/adolescent death/disappearance are subject to multiple local agencies’ investigations (law enforcement, child protective services) and may receive assistance from national entities (NCMEC, Amber alerts etc.) all working simultaneously to increase identifications.

Older adults may also have a similar system of societal surveillance related to where they die and who is looking for them. According to Muramatsu and colleague’s [67] study on place of death for older Americans, 33% died in nursing homes, <25% died at home, and the remainder died in either hospice or hospital care. Thus, the largest majority of older adults have a direct relationship to surveillance via care. Older adults often have consistent care appointments, or relationships with targeted programs like meals on wheels that surveille well-being in some metric. In addition, for older adults that may go missing, the American Silver Alert system is a public notification system countrywide that issues alerts on missing seniors, especially those that may have dementia or other cognitive impairments. Together these systems of surveillance amount to an established network of social programs that may work collaboratively to increase the identification of both adolescents and older adults in the United States.

In regard to decedent race and/or ethnicity, Aim 1 found that the pooled sample yielded no significant relationship between identification rates and decedent race and/or ethnicity s, but UNT’s agency-specific test was significant (Table 5), and correspondence analysis indicated that the difference was driven by the higher identification rates for White decedents (81%) compared with Hispanic decedents (42%). UNT has more young adult, Hispanic male cases than both NYC and FBI, which is a common demographic of decedents believed to have died during an attempt to cross the U.S.-Mexico border, and multiple studies have documented the difficulties in successfully identifying decedents within this context [68–74].

Martinez and colleague’s work [70] examining Pima County Office of the Medical Examiner (PCOME) case data, highlighted a relationship between decedent demographics and identification rates, namely that as the number of non-Mexican, Central American individuals crossing the border increased in recent years, so too did the number of unidentified remains cases. The authors posited that poverty, consulate resource limitations, and the strategies of human smugglers along the border all increase the complexity of the investigation and create disparities in the odds of a successful identification of decedents from Central America as compared to more traditional migration regions like Mexico. Hughes et al. [69] and Algee-Hewitt et al. [68] found a similar identification disparity in PCOME forensic casework related to the ancestry of deceased individuals along the U.S.-Mexico border, in that identified migrants tended to have more European ancestry, while unidentified migrants tended to have less European ancestry. Importantly, the identification bias was unlikely to be attributed to any direct action (or inaction) by the investigators, as these particular ancestry estimates were unknown to the investigators (and the forensic anthropologists working the cases) at the time of the investigations; the ancestry estimations were instead derived from CODIS markers and/or craniometric analyses by the authors well after most of the cases had undergone their initial investigations.

These studies represent a context where all the cases were considered to be a part of a vulnerable demographic—undocumented individuals migrating across the U.S. Mexico border—yet there were still biases in the odds of being identified. Similar to Martínez et al. [70], these studies proposed that structural vulnerabilities for individuals migrating from Mexico have emerged from the economic, political, and social processes in Mexico and the United States such that certain populations (in this case, more rural, southern Mexico, and/or Indigenous) were less often able to access and/or trust government and non-governmental social services that aid in the identification of missing loved ones, such as the collection of DNA family reference samples or filing of missing persons reports, which are key to making identifications. Indeed, this finding is echoed by research focusing on how sociohistoric factors and structural vulnerabilities play a role in investigation outcomes for undocumented Latinx individuals [68, 71]. These previous studies, along with the present study’s findings that only the UNT sample exhibited race and/or ethnicity disparities in identification rates, strongly suggest that context-specific dynamics can potentially influence the odds of an identification. As a result, the large number of U.S-Mexico border cases may inherently negatively impact the identification rates for UNT’s Hispanic subsample. However, data on the exact ratio of UNT Hispanic cases from NamUs Region 4 (which includes Texas) that are related to the border crisis was not available for the present study, and therefore we cannot conclude that these trends related to identification rates are solely a result of the United States-Mexico border context. More research on these trends may elucidate the most significant sources of the differing identification rates.

### Does agency context matter?

Agency differences in identification trends highlight that the practice (and its outcomes) of forensic anthropology will inherently vary according to the context and mandates of each agency. This has important implications for research and casework, in that there may not be nation-wide trends, or, while inferable, may not be useful for understanding the ground-level practice of forensic anthropology in the United States. In this study, we have worked to incorporate models that can investigate identification trends while simultaneously accounting for agency-specific nuances. Here, we review some of the factors that may contribute to the differences we observed in identification trends across the agencies.

We found that NYC exhibited outcomes that suggest a consistent level of high performance that merited deeper examination. NYC had the greatest identification rate and exhibited a positive significant effect of reporting the biological profile on identification rate. There are substantial differences in the three agencies’ sample contexts and investigative approaches that may contribute to these trends. Both UNT and FBI forensic anthropology services span almost the entire country and they provide consultations to medical examiner/coroner and law enforcement agencies nationwide, and it is possible that the FBI and UNT may be getting a subset of cases that are inherently more difficult to identify (i.e., incomplete skeletonized remains). The FBI and UNT datasets primarily include forensic anthropology cases which are under external jurisdiction, and therefore UNT and FBI’s involvement in their cases is limited to the services requested by the originating agency. In contrast, the NYC sample in general represents cases from New York City, and within the jurisdiction of the NYC OCME. Thus, NYC OCME is in complete control of the entire investigative process, with the forensic anthropology unit deeply embedded in the investigations. These are key differences in that the results here are not truly a reflection of UNT and FBI as investigative agencies. Instead, the trends reported here for UNT and FBI reflect nation-wide trends of investigative agencies that use FBI and UNT services. Because data on the investigative agencies with jurisdiction for the cases included in the FBI and UNT samples are not available, we are unable to discuss the agency-specific practices and contexts. In contrast, because the NYC sample is by and large specific to NYC OCME jurisdiction, we focus here on the practices and investigation processes at NYC OCME in relation to their observed identification trends. We review additional factors that may contribute to identification trends across agencies in the Supporting Information.

## Conclusions

The discipline of forensic anthropology has recently begun to more deeply reflect on the impact of our practices. Here, we contribute to this essential work by examining how forensic anthropology estimations of biological profile components and decedent demographics are related to identification trends. The results presented in this study indicate that for forensic anthropology cases: i) there is limited consistent evidence that decedent sex, age, and race and/or ethnicity are related to identification trends in the pooled United States sample; ii) when substantial differences (e.g. medium effects) do occur, they appear to be more agency-specific and related to the particular contexts of that agency; iii) there is no consistent evidence for discrepancies in the duration of an investigation based on a decedent’s sex or age, while race and/or ethnicity need more investigation; iv) forensic anthropological estimations of sex, age, and ancestry appear to have a small positive relationship with improved identification rates for casework.

Importantly for the present sample, forensic anthropology cases do not appear to exhibit consistent identification disparities other than those already known (such as the difficulty of identifying undocumented migrants). These results in no way negate the extensive documentation of racial bias in the criminal justice system, the possibility of delayed/deprioritization of particular investigations by law enforcement, nor the structural vulnerabilities specifically related to marginalized groups be it related to gender, race, ethnicity, socioeconomic status, or other factors. Furthermore, while our study includes cases from all NamUs regions in the U.S., our results may not necessarily determine whether disparities exist or impact identification investigations in the general U.S. practice of forensic anthropology, given that agency context and practice likely play the biggest roles in identification trends. In fact, the three study samples selected for this study reflect the best-case-scenario, in that these three agencies are state-of-the art in terms of resources, technology and quality assurance, and have multiple full-time forensic anthropologists on staff. While the best-case-scenario to practice forensic anthropology, these study samples may not be representative of all coroner and medical examiners offices in the U.S. Therefore, the current findings are a first step, and indeed, indicate that when intentional measures are taken to utilize the best investigative practices currently available, decedent demographics of deceased individuals have limited effects on reducing the chances of being identified and reconnected with their loved ones. Conversely, the findings specific to UNT’s identification rate disparities between Hispanic and White decedents suggest that some investigative contexts, such as the crisis at the U.S.-Mexico border, have hurdles that may not be fully overcome even in an agency with robust investigative means.

The findings presented here provide ample opportunity on which to expand further. Studies have hypothesized how other contextual factors that contribute to structural vulnerability and marginalization, such as gender identity, sexual preferences, race, citizenship, stigma, and socioeconomic status may contribute to identification disparities beyond the biological profile [5, 20, 69, 75–78]. While this study found that decedent sex, age, and race/ethnicity have a limited relationship with identification trends for the study samples, other factors like socioeconomic status (SES), which are an important contributor to marginalization in the United States [79], was not taken into account. Unfortunately, there does not appear to be systematic data of the demographics and SES of individuals within the United States medicolegal death investigation system. However, based on research examining mortality in the United States more generally, SES appears to be the leading factor contributing to mortality overall, with sex and race being important factors affecting mortality within SES groups [e.g., 80–82].

Within the context of the conversation around whether and/or how to practice ancestry estimation, the results of this study offer a single data point on whether the race of a decedent is related to the odds of being identified and cannot speak to whether estimating ancestry is harming BIPOC communities within other contexts of the identification process or the lived experiences of these communities at large. Thus, the conversation is ongoing and the future discussions on ancestry estimation must still include much of what was originally called for by Bethard and DiGangi [1] as well as Stull and colleagues [2].

Using over 1,200 unidentified and identified forensic anthropology cases from three United States agencies, this study provides the first comprehensive, data-driven insight into the specific relationships between decedent data, biological profile estimations, and identification status for United States forensic anthropology cases. Even with this data, our analyses and findings are limited to the demographics that had adequate sample sizes or appropriate approaches for making inferences about the data. As such, key groups (e.g., multiracial, transgender individuals) were unable to be analyzed, and future studies will greatly benefit from a more inclusive dataset. We also acknowledge that the kind of data used in this study, namely retrospective case metrics from U.S. investigative agencies, are only one contribution to these important discussions in our discipline and is not without its own limitations. Social science approaches like ethnography will greatly contribute in ways that the data presented here cannot, and would certainly be better at measuring (de)prioritization than the proxy variables implemented here. As additional research incorporating comprehensive data continues to be developed, our understanding of biases related to the biological profile and other relevant factors will be improved, and our strategies for addressing those of significance can be refined.

## Supporting information

S1 File (DOCX)
