# Supplementary material for: Investigating identification disparities in forensic anthropology casework
Source: PLoS One. 2023 Nov 1;18(11):e0290302. doi: 10.1371/journal.pone.0290302 (PMC10619877; doi:10.1371/journal.pone.0290302)
Supplement: S1 File — (DOCX) [file pone.0290302.s001.docx]

**Supporting Information**

**Materials and Methods**

**Case standardization**

**Dataset for Aims 1 and 3.**

Aim 1 is concerned with the overall investigative outcomes for various demographics, *regardless of the specific demographic information provided or not provided by the forensic anthropologist.* Therefore, for Aim 1, decedent demographic data drawing on both known and estimated demographic data is employed. Aim 3 is interested in the relationship between case resolution (i.e. successful identification of the decedent) and the presence or absence of estimations of sex, age, and ancestry in a forensic anthropology report. Therefore similar to Aim 1, Aim 3 used the dataset which incorporated both known and estimated demographic data. For Aims 1 and 3, the variables of “decedent sex” and “decedent age” were generated from the three agencies’ datasets using a similar approach. If the decedent was identified, then the known sex and age were used as “decedent sex” and “decedent age” for this study. If there was an estimate produced by the forensic anthropologist for either sex or age, this was ignored for identified decedents with available decedent information. If an identified case did not have a documented sex for the decedent, then the forensic anthropology estimation of sex was used. All available decedent sex data for identified cases resulted in one of two sex designations, either male or female.

For those cases *without* known decedent information on sex or age (all unidentified cases and some identified cases), then the forensic anthropology estimates for sex and age were used to represent the decedent sex and age data for this study, when present. Indeterminate sex and age estimations resulted in no decedent sex or decedent age data extraction for that case. Sex estimations that used the qualifiers “possible” or “probable” were considered as the sex insinuated by either qualifier (e.g., “probable female” = female). No forensic anthropology estimations reported multiple designations, or anything other than male or female sex categories (besides previously mentioned qualifiers). Therefore, when referring to “decedent sex” and “decedent age” in this study, these represent a composite inference that includes either the known (for identified cases) or estimated sex and age for that particular decedent.

In choosing the binary categories for decedent sex, and common United States social race and ethnicity categories, this analysis may lack the nuanced reality of how the decedents may have self-identified. We acknowledge this as a limitation in this study but are unable to make assumptions beyond the information that is provided in the datasets, which adhere to these categories. The selection of the categories and labels used in the present study were chosen to most accurately reflect those used in casework so inferences can be made that directly relate to and represent casework.

Decedent age data was represented as both age interval data (for anthropological estimates of age) and exact age data (the known age for identified decedents). We used the age data to categorize each decedent into a standard age cohort [1]: Juvenile (fetal to 11 years), Adolescents (12 to 19 years), Young Adults (20-34 years), Middle Adults (35-49 years), and Older Adults (50+ years). The decision to represent individuals younger than 12 years of age as a single cohort was due to the small sample size. Decedents with a known age (only available in identified cases) were placed into the cohort that corresponded with their age. Decedents for which an exact age was unknown (unidentified cases and identified cases missing age information) were placed into their respective age cohort using forensic anthropology estimations of age with the following parameters. If the entire estimated age range fell into a single age cohort, then the decedent was assigned to that cohort (e.g., “20-24 years of age” = Young Adult). Alternatively, when the estimated age range spanned two or more cohorts (e.g., 18-24 years of age = Adolescent *and* Young Adult), the average for the estimated age interval was calculated ([minimum age + maximum age]/2) and used as the point estimate for assigning that decedent to a single age cohort (e.g., [18 +24]/2 = 21 years of age = Young Adult). Cases were considered “inconclusive” if the forensic anthropologically estimated age range was too general (e.g., “adult”) or spanned more than four decades. Individuals with an “inconclusive age” were excluded from an age cohort assignment and any associated statistical analyses that require an age cohort assignment.

We tested the utility of this approach using our sample of identified decedents for which age was known and that also had corresponding estimated age ranges (*n* = 320). The residuals between the two (mean estimated age - decedent known age) were calculated, and the median residual was 0.5 years, with an interquartile range of -2.5 years to 4.0 years. Given that the majority of estimated age ranges classified decedents into a single age cohort, and the residuals are small compared to the age intervals (spanning multiple decades), the proposed approach of using the average estimated age to assign cases to a decedent age cohort was deemed acceptable.

Lastly for Aims 1 and 3, the variable “decedent race and/or ethnicity” was generated similarly to decedent sex and age data, but with several additional considerations. In almost all cases from all three agency datasets, the ancestry estimated by forensic anthropology examination was reported using standard United States race and ethnicity labels (e.g., Black, White) instead of geographic indicators like “African” or “European.” Similarly, in almost every case with known decedent information, the decedent data was reported using standard United States race and ethnicity census labels. The consistent use of standard United States race and ethnicity classifications made merging datasets more straightforward, resulting in the following groups studied: Asian, African American or Black, Hispanic, Native American, White, and a final category labeled “Additional Races and/or Ethnicities” to represent those not comprehensively represented by one of the above groups.

When decedent race and/or ethnicity needed to be inferred (for those cases without known decedent data but with available forensic anthropology ancestry estimates), we used the approach laid out by Hughes, Juarez (2) which took into account the forensic anthropology ancestry estimations. Therefore, in this study when referring to “decedent race and/or ethnicity”, this data represents a composite inference that includes either the known race or ethnicity (for identified individuals) or estimated ancestry for that particular decedent. When the forensic anthropology ancestry estimation was solely relied upon to infer decedent race and/or ethnicity, there were a handful of times when more than one descriptor was provided by the forensic anthropologist. Because there is limited research on the relationship between complex or multiple ancestry interpretations and actual decedent race identity , we cannot confirm whether such estimates should be interpreted as multiracial, or as representing an individual whose skeletal variation falls within a range of ambiguity for the applied ancestry estimation methods, and/or is from a group that is not readily represented in our methods’ reference samples. Because a definitive assessment was not provided and could not be readily interpreted, cases with multiple ancestry estimations were not assigned to a decedent race and/or ethnicity category (instead referred to as “Inconclusive” decedent race and/or ethnicity) and thus excluded from statistical analyses where a definitive decedent race and/or ethnicity category was required.

With the approach outlined above, individuals in the “Additional Races and/or Ethnicities” category only included identified individuals with an available description of the decedents’ race(s) and/or ethnicity(ies). No unidentified cases could populate the “Additional Races and/or Ethnicities” category due to similar reasons listed above, in that we cannot readily interpret a nondescript ancestry estimator (e.g., “Other”) or multigroup ancestry estimation (e.g. “Native American and African ancestries”) to a decedent race and/or ethnicity category. Indeed, Winburn and Algee-Hewitt (3) and Hughes, Juarez (2) highlight the difficulties in assessing accuracy for these particular ancestry estimations, and the low accuracy rates when attempted. Given these limitations, we do not place any individual into the “Additional Races and/or Ethnicities” category if there is only a forensic anthropology estimation of ancestry, which inherently skews this subsample to only include identified decedent cases with available race and/or ethnicity data. In contrast, the “Inconclusive” decedent race and/or ethnicity subsample includes both unidentified individuals with complex or ambiguous ancestry estimations as well as identified individuals with no information on decedent race and/or ethnicity *and* did not have definitive ancestry estimates. Therefore, neither the “Inconclusive” nor the “Additional Races and/or Ethnicities” categories can be analyzed in the present study when examining trends of decedent race and/or ethnicity. Ancestry, which may be better described as population affinity, is the application of geographically-structured skeletal variation that corresponds with both deep and recent histories of microevolutionary processes of populations across the world. As such, ancestry corresponds with population histories, which in turn correspond with geography and time. Importantly, while estimated ancestry is fundamentally different from the socially-constructed concept of race, race borrows on heritage which is also linked to geography and population movements. Race also draws on phenotypes (e.g. skin color) that are geographically structured, similar to skeletal variation. Thus, ancestry and race are spuriously correlated, as they both covary with geography, and forensic anthropologists engage this correlation between skeletal variation and societal concepts of race when attempting to translate a decedent’s estimated ancestry to social race. Importantly, this correlation between race and ancestry, does not provide grounds for the justification of biological races. Furthermore, forensic anthropologists’ employment of this correlation in their investigations does not support the notion of race as a biological concept, but instead acknowledges the spurious correlation as a tool that can help narrow down investigative leads.

**Dataset for Aim 2.** Aim 2 is primarily concerned with how the *specific* demographic information (e.g. white middle-aged female) provided or not provided to the case investigator in the forensic anthropology report may be related to variation in investigation efforts and identification success. Ergo, the decedent demographic information employed for analyzing Aim 2 is derived solely from the *estimated* decedent sex, age, and ancestry by forensic anthropologists. Standardizing the forensic anthropological estimates of sex, age, and ancestry in large part conformed to the approaches outlined above for Aims 1 and 3 but without incorporating considerations of the *known* demographic data. The key exception where the data standardization differs from Aims 1 and 3 is for forensic anthropology ancestry estimations. Ancestry estimations that included multiple descriptors for a single individual were incorporated into the dataset labeled as “Multiple.” Thus, the Inconclusive category for Aim 2 also differs from Aim 1 and 3 in that for Aim 2 it is only comprised of cases for which the ancestry estimation was left blank or listed as some version of inconclusive or indeterminate.

A final consideration in our approach to extracting decedent demographic data is the use of forensic anthropology estimates of the sex, age, and ancestry to represent actual decedent demographic data. In order to compare decedent demographic trends between identified and unidentified individuals, it is necessary to rely on the forensic anthropological estimates for all unidentified decedent cases, as well as the handful of identified individuals that were missing information regarding the decedent’s known age, sex and/or ancestry. The assumption that we make for the present study is that the forensic anthropologists’ accuracy for estimating decedent sex, age, and race and/or ethnicity is high for those cases, and thus inferred decedent demographics adequately reflect decedent data. Previous studies have shown that age, ancestry, and sex estimations are over 90% accurate [2, 4-6]. Furthermore, when inaccuracies occurred, no statistically significant differences in rates of inaccuracies were found among particular subsamples for age [2], sex [5], or ancestry [2, 6]. The only exception is that females were more likely to have their sex incorrectly estimated compared to males [2]. However, even if it is assumed that this sex trend holds for the present study, it would minimally impact the decedent sex allocations because the frequency of inaccurate estimations for sex are extremely small, between 0-5% [2, 4, 5].

**Results**

**Expanded results for Aim 2.**

**Identification Rates**

Chi-Square tests were performed to examine variation of investigation success (decedent identified or not identified) across estimated decedent demographic data (sex, age at death, and ancestry).

Pooled analysis of male and female identification success yielded significant differences, with females exhibiting greater identification success than males. The analysis comparing all age cohorts was also significant, and similar to the Aim 1 results, two age cohort clusters emerge, with Young and Middle Adult cohorts being less often identified (40-42% cases are resolved), while Adolescent, Juvenile, and Older Adult cohorts clustered with higher case resolution rates of (52-55%).

Pooled analysis of estimated decedent ancestries of Asian (*n* = 28), Black (*n* = 178), Hispanic (*n* = 240), White (*n* = 354), and those with multiple descriptor labels (*n* = 166) yielded significant differences in investigative success. Similar to the results in Aim 1, decedents estimated as White and Black had the greatest investigative success with 54% and 53% of cases identified, respectively. Clustered with lower investigation success rates (39%-41%) are decedents estimated to be Asian, Hispanic, or multiple labels.

The Agency-specific tests for difference in investigative success for estimated males versus females were not significant for FBI, NYC or UNT. Agency-specific analyses of investigative success related to estimated decedent age included all but juvenile age cohorts. Both FBI and NYC yielded no significant differences in investigative success for Adolescent, Young Adult, Middle Adult and Older Adult cohorts. In contrast, UNT exhibited significant differences, with a notable reduction in investigative success for the Young Adult cohort at 30% identified cases compared with all other age cohorts (49%-57% successfully identified). Agency-specific analyses of investigative success related to estimated decedent ancestry yielded significant differences in two agencies, NYC and UNT. The estimated ancestry groups with adequate samples included Black, Hispanic, White, and those with multiple descriptors. For NYC, the significance is largely driven by the fact that all 17 decedents estimated as multiple ancestry descriptors remain unidentified, while decedents estimated as Black, Hispanic and White cases are more comparable with 67-75% investigative success. For UNT, the significant differences in are consistent with those found in the similar Aim 1 analysis, such that decedents estimated to be White (67% success) are nearly twice as often identified as decedent estimated to be Hispanic (36% case success), while those estimated to be Black (55% case success) and as multiple descriptors (47%) are intermediate.

**S1 Table. Chi-Square tests of identification success variation and estimated decedent demographic data. Pooled and agency-specific tests performed.** Shaded cells indicate significance of adjusted *p*-value.

| Test | n, df | Likelihood Ratio | Adjusted *p* | Cramer’s *V*, 95% CI | Effect Size |
| --- | --- | --- | --- | --- | --- |
| ID Status x estimated sex (pooled) | 1141, 1 | 3.46 | 0.036 | 0.05, 0.005-0.114 | Negligible |
| ID Status x estimated age (pooled) | 1000,4 | 11.87 | 0.027 | 0.11, 0.066-0.186 | Small |
| ID Status x estimated ancestry (pooled) | 966,4 | 16.942 | 0.006 | 0.13, 0.087-0.200 | Small |
| **Agency Specific Results Below** |  |  |  |  |  |
| ID Status x estimated sex (FBI) | 494,1 | 1.73 | 0.189 | 0.06, 0.004-0.149 | Negligible |
| ID Status x estimated sex (NYC) | 330,1 | 0.50 | 0.480 | 0.04, 0.002-0.144 | Negligible |
| ID Status x estimated sex (UNT) | 317,1 | 1.71 | 0.334 | 0.04, 0.002-0.142 | Negligible |
| ID Status x estimated age (FBI) | 432,3 | 6.84 | 0.189 | 0.13, 0.057-0.239 | Small |
| ID Status x estimated age (NYC) | 257,3 | 3.53 | 0.475 | 0.12, 0.053-0.254 | Small |
| ID Status x estimated age (UNT) | 300,3 | 12.84 | 0.019 | 0.20, 0.124-0.330 | Small |
| ID Status x estimated ancestry (FBI) | 410,3 | 5.39 | 0.189 | 0.11, 0.047-0.213 | Small |
| ID Status x estimated ancestry (NYC) | 262,3 | 38.43 | 0.0003 | 0.36, 0.282-0.451 | Medium |
| ID Status x estimated ancestry (UNT) | 266,3 | 12.68 | 0.019 | 0.22, 0.122-0.354 | Small |

**Investigation Duration**

The Kruskal-Wallace rank sum test was performed to compare investigation duration (number of years to identification) among UNT decedents estimated to be Hispanic, (*n* = 53), White (*n* = 17) and those that had multiple ancestry labels associated with them (*n* = 20). No significant difference in distributions among the three tests were found (S2 Table) when examining cases from 2012-2021. A similar analysis was performed for cases from 2014-2021comparing decedents estimated to be Hispanic, (*n* = 42), White (*n* = 12) and those that had multiple ancestry labels associated with them (*n* = 18) with no significant differences in investigative duration distributions (S1 Table).

Comparing investigation duration for UNT decedents estimated to be male (*n* = 102) and female (*n* = 103) were conducted with no significant differences. Similarly, no significant differences were found in investigation duration among Young Adults (*n* = 29), Middle Adults (*n* = 71), and Older Adults (*n* = 14).

**S2 Table. Kruskal-Wallace rank sum results for testing investigative duration distribution similarities among demographic factors.** Shaded cells indicate significance of adjusted *p*-value.

| Estimated Demographic Factor | n | Test Statistic, df | Adjusted *p* |
| --- | --- | --- | --- |
| Sex | 133 | 4.505, 1 | 0.079 |
| Age | 114 | 2.00, 2 | 0.429 |
| Ancestry (cases 2012-2021) | 90 | 2.489, 2 | 0.403 |
| Ancestry (cases 2014- 2021) | 72 | 0.6936, 2 | 0.707 |

**Multivariate Analysis**

A mixed effects logistic regression was used to explore the relationship between investigation success (decedent identified or not identified) and estimated decedent demographic data (sex, age at death, and ancestry) in a multivariate context. Odds ratios (S3 Table) and 95% identification probabilities (S1 Fig).

**S3 Table. Odds ratios of identification success estimated from the multivariate mixed effect logistic regression model using forensic anthropologists’ estimates of sex, age, and ancestry.** The reference categories (odds ratio = 1) are female, adolescent, and White.

| **Predictors** | **Odds Ratio** | **CI** | **p** |
| --- | --- | --- | --- |
| **Estimated sex** |  |  | 0.724 |
| **Male** | 0.75 | 0.49 – 1.14 | 0.173 |
| **Estimated age category** |  |  | 0.988 |
| **Young Adult** | 1.02 | 0.43 – 2.42 | 0.971 |
| **Middle Adult** | 1.06 | 0.45 – 2.52 | 0.889 |
| **Older Adult** | 1.46 | 0.54 – 3.96 | 0.461 |
| **Estimated ancestry** |  |  | 0.239 |
| **Asian** | 0.34 | 0.10 – 1.16 | 0.086 |
| **Black** | 0.73 | 0.46 – 1.17 | 0.193 |
| **Hispanic** | 0.44 | 0.27 – 0.69 | < 0.001* |
| **Combinations** | 0.24 | 0.03 – 1.95 | 0.184 |

**S1 Fig.** **Estimated probabilities of identification for the combination of estimated biological profile components (sex and ancestry).** Note the overlaps among different groups suggest the effects of estimated biological profile components on identification probabilities are small.


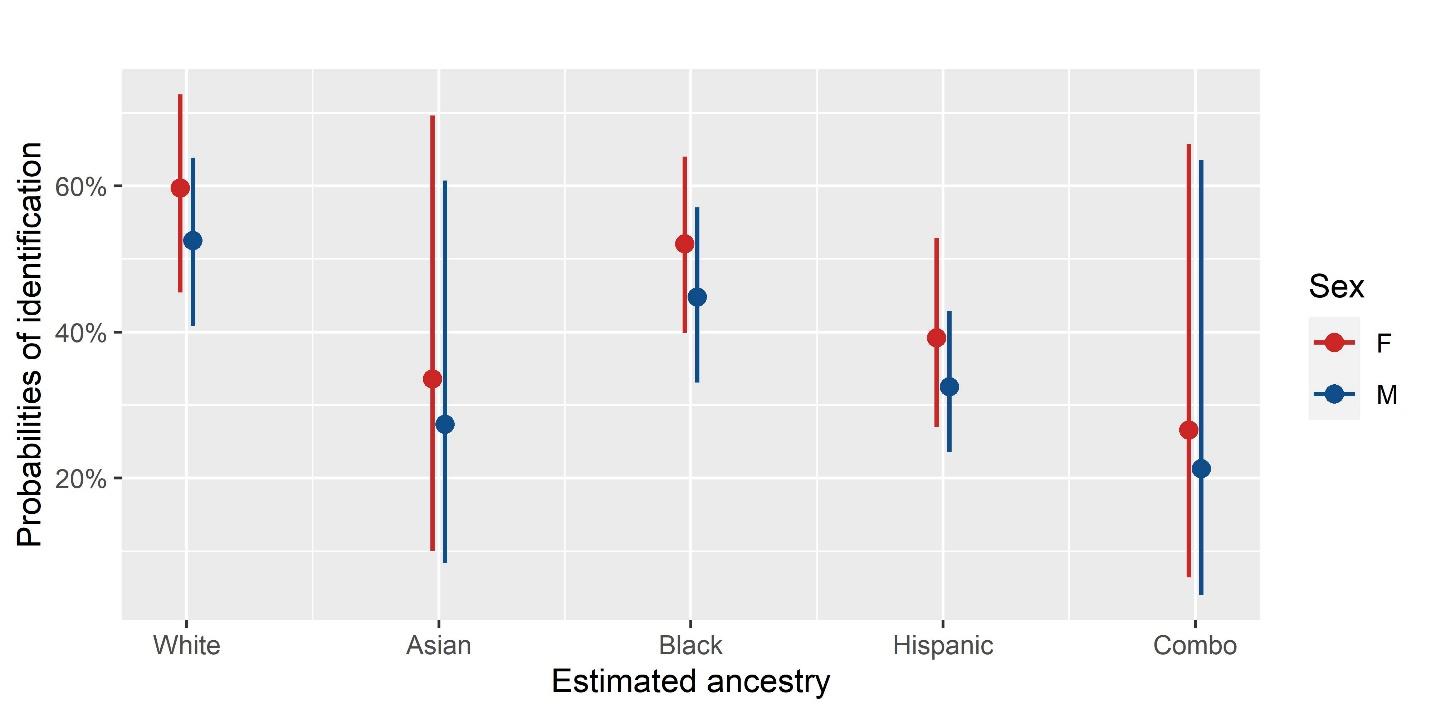


**Discussion**

**Expanded Discussion: Does Agency Context Matter?**

The NYC OCME is a large local agency with numerous in-house investigative resources, such as a Forensic Anthropology Unit (FAU), dedicated Identifications and Family Outreach Units, access to local and national fingerprint databases, the largest public DNA Laboratory in the world, and in-house collaboration with an NYPD Missing Persons Morgue Detective Unit. Furthermore, forensic anthropology is well-established at the NYC OCME and if a forensic anthropological approach is useful for any unknown case of skeletonized, decomposed, or even visually identifiable remains, then it will be implemented. Forensic anthropologists at the NYC OCME are well-integrated into the broader investigative process, working closely and directly with the agency’s dedicated Identifications Unit, as well as other City agencies, and other local and national governmental and nongovernmental agencies that can assist in missing and unidentified persons investigations. As an integral part of the identification investigation, NYC OCME forensic anthropologists also upload and actively manage all New York City unidentified and unclaimed remains cases in NamUs and conduct their own searches and follow-up for scientific comparison to missing persons. In addition to these resources, several years ago the NYC OCME created a multidisciplinary Identification Review Committee where members of various investigational departments, including the FAU, attend a monthly meeting to discuss complex identification investigations and examine additional avenues for identification of unknown persons. As such, the increased identification rates are likely a reflection of the development and continued access to numerous investigative resources within the agency.

The agency-specific contexts and complexities of missing and unidentified persons cases cannot be overemphasized. Indeed, missing persons reports, or the lack thereof, are likely a limiting factor for all the identification trends reported here, regardless of agency. Without missing person reports, the generation of investigative leads, and the collection and upload of associated family DNA reference samples are not possible. Some of the obstacles to collecting missing persons reports and family reference samples may be overcome by working with non-governmental organizations serving as liaisons for families of the missing, such as has been recommended and in some cases implemented for the missing migrant context along the United States-Mexico border [7-9], as well as programmatic interventions for high-risk groups [10]. However, reticence to trust law enforcement and governmental agencies may remain a reality for some demographic groups [11, 12]. Alternatively, other research has indicated that Latinx in Philadelphia and Los Angeles express ambivalence towards police and in general consider them trustworthy [13]. As such, community mistrust may only be one potential factor that affects family reference sample participation and/or the filing of missing persons reports. Beyond trust, there are other barriers that may prohibit individuals from reporting missing persons that still remain. Such barriers include lack of knowledge by a friend or family member that a loved one has gone missing (often in cases of highly transient individuals), lack of knowledge about resources for where or how to report a missing loved one [14], as well as the resistance of some law enforcement agencies to collect reports of missing individuals who they incorrectly believe to have disappeared voluntarily or because they believe the case is outside their jurisdiction [15]. Future investigative approaches require the continued development of nuanced and targeted interventions to gain vital information from families and the increased integration of forensic anthropologists into the identification investigation.

**References**

1. Buikstra JE, Ubelaker DH. Standards for data collection from human skeletal remains. 1994.

2. Hughes CE, Juarez C, Yim A-D. Forensic anthropology casework performance: Assessing accuracy and trends for biological profile estimates on a comprehensive sample of identified decedent cases. J Forensic Sci. 2021;66(5):1602-16. doi: <https://doi.org/10.1111/1556-4029.14782>.

3. Winburn AP, Algee-Hewitt B. Evaluating population affinity estimates in forensic anthropology: Insights from the forensic anthropology database for assessing methods accuracy (FADAMA). J Forensic Sci. 2021;66:1210-9. doi: <https://doi.org/10.1111/1556-4029.14731>.

4. Parsons HR. The Accuracy of the Biological Profile in Casework: An Analysis of Forensic Anthropology Reports in Three Medical Examiners' Offices: University of Tennessee, Knoxville; 2017.

5. Thomas RM, Parks CL, Richard AH. Accuracy Rates of Sex Estimation by Forensic Anthropologists through Comparison with DNA Typing Results in Forensic Casework. J Forensic Sci. 2016;61(5):1307-10. doi: <https://doi.org/10.1111/1556-4029.13137>.

6. Thomas RM, Parks CL, Richard AH. Accuracy Rates of Ancestry Estimation by Forensic Anthropologists Using Identified Forensic Cases. J Forensic Sci. 2017;62(4):971-4. doi: <https://doi.org/10.1111/1556-4029.13361>.

7. Hughes CE, Algee-Hewitt BFB, Reineke R, Clausing E, Anderson BE. Temporal Patterns of Mexican Migrant Genetic Ancestry: Implications for Identification. Amer Anthrop. 2017;119(2):193-208. doi: <https://doi.org/10.1111/aman.12845>.

8. Reineke R, Monterroso MM, editors. Science as a Human Right: Using DNA to Identify Missing Migrants. 2018 AAFS Annual Scientific Meeting; 2018; Seattle, WA.

9. Spradley MK. Toward estimating geographic origin of migrant remains along the United States–Mexico border. Annals of Anthropological Practice. 2014;38(1):101-10. doi: <https://doi.org/10.1111/napa.12045>.

10. Katsanis SH, Felini M, Kim J, Minear M, Chandrasekharan S, Wagner JK. Perspectives of Women in Prostitution diversion program on DNA Collection for a High-Risk DNA Database. International Journal of Criminal Justice Sciences. 2017;12(1):111-28. doi: 10.5281/zenodo.345720.

11. Cole LM, April K, Trinkner RJ. The Black and White Reality: Historical and Post-Ferguson Era Perspectives on Public Attitudes Toward the Police. In: Miller MK, Bornstein BH, editors. Advances in Psychology and Law: Volume 5. Cham: Springer International Publishing; 2020. p. 267-99.

12. Theodore N, Habans R. Policing immigrant communities: Latino perceptions of police involvement in immigration enforcement. Journal of Ethnic and Migration Studies. 2016;42(6):970-88. doi: 10.1080/1369183X.2015.1126090.

13. Armenta A, Rosales R. Beyond the Fear of Deportation: Understanding Unauthorized Immigrants’ Ambivalence Toward the Police. Am Behav Sci. 2019;63(9):1350-69. doi: 10.1177/0002764219835278.

14. Quinet K. The Missing Missing:Toward a Quantification of Serial Murder Victimization in the United States. Homicide Stud. 2007;11(4):319-39. doi: 10.1177/1088767907307467.

15. Reineke R, Halstead C. Identifying dead migrants: Examples from the United States–Mexico border. In: Laczko F, Singleton A, Black J, editors. Fatal Journeys. 3. Geneva, Switzerland: International Organization for Migration 2017. p. 77-98.
